# *Debaryomyces hansenii* supplementation in low fish meal diets promotes growth, modulates microbiota and enhances intestinal condition in juvenile marine fish

**DOI:** 10.1186/s40104-023-00895-4

**Published:** 2023-07-09

**Authors:** Ignasi Sanahuja, Alberto Ruiz, Joana P. Firmino, Felipe E. Reyes-López, Juan B. Ortiz-Delgado, Eva Vallejos-Vidal, Lluis Tort, Dariel Tovar-Ramírez, Isabel M. Cerezo, Miguel A. Moriñigo, Carmen Sarasquete, Enric Gisbert

**Affiliations:** 1 Aquaculture Program, Institute of Agrifood Research and Technology (IRTA), La Ràpita, 43540 Spain; 2 Centro de Biotecnología Acuícola, Universidad de Santiago de Chile, Santiago, Chile; 3 Instituto de Ciencias Marinas de Andalucía (ICMAN-CSIC), Avda. República Saharaui nº 2, Campus Universitario Río San Pedro, Puerto Real, Cádiz, 11510 Spain; 4 Núcleo de Investigaciones Aplicadas en Ciencias Veterinarias y Agronómicas, Facultad de Medicina Veterinaria y Agronomía, Universidad de Las Américas, Santiago, Chile; 5 Department of Cell Biology, Physiology, and Immunology, Universitat Autonoma de Barcelona, Barcelona, Spain; 6 Centro de Investigaciones Biológicas del Noroeste SC, CIBNOR, La Paz, México; 7 Department of Microbiology, Instituto de Biotecnología Y Desarrollo Azul (IBYDA), Faculty of Sciences, University of Malaga, 29010 Malaga, Spain; 8 SCBI, Bioinformatic Unit, University of Malaga, 29590 Malaga, Spain

**Keywords:** *Debaryomyces hansenii*, Intestine condition, Low fish meal diet, Microbiota, Transcriptomics, Yeast probiotic

## Abstract

**Background:**

The development of a sustainable business model with social acceptance, makes necessary to develop new strategies to guarantee the growth, health, and well-being of farmed animals. *Debaryomyces hansenii* is a yeast species that can be used as a probiotic in aquaculture due to its capacity to i) promote cell proliferation and differentiation, ii) have immunostimulatory effects, iii) modulate gut microbiota, and/or iv) enhance the digestive function. To provide inside into the effects of *D. hansenii* on juveniles of gilthead seabream (*Sparus aurata*) condition, we integrated the evaluation of the main key performance indicators coupled with the integrative analysis of the intestine condition, through histological and microbiota state, and its transcriptomic profiling.

**Results:**

After 70 days of a nutritional trial in which a diet with low levels of fishmeal (7%) was supplemented with 1.1% of *D. hansenii* (17.2 × 10^5^ CFU), an increase of *ca.* 12% in somatic growth was observed together with an improvement in feed conversion in fish fed a yeast-supplemented diet. In terms of intestinal condition, this probiotic modulated gut microbiota without affecting the intestine cell organization, whereas an increase in the staining intensity of mucins rich in carboxylated and weakly sulphated glycoconjugates coupled with changes in the affinity for certain lectins were noted in goblet cells. Changes in microbiota were characterized by the reduction in abundance of several groups of Proteobacteria, especially those characterized as opportunistic groups. The microarrays-based transcriptomic analysis found 232 differential expressed genes in the anterior-mid intestine of *S. aurata*, that were mostly related to metabolic, antioxidant, immune, and symbiotic processes.

**Conclusions:**

Dietary administration of *D. hansenii* enhanced somatic growth and improved feed efficiency parameters, results that were coupled to an improvement of intestinal condition as histochemical and transcriptomic tools indicated. This probiotic yeast stimulated host-microbiota interactions without altering the intestinal cell organization nor generating dysbiosis, which demonstrated its safety as a feed additive. At the transcriptomic level, *D. hansenii* promoted metabolic pathways, mainly protein-related, sphingolipid, and thymidylate pathways, in addition to enhance antioxidant-related intestinal mechanisms, and to regulate sentinel immune processes, potentiating the defensive capacity meanwhile maintaining the homeostatic status of the intestine.

**Supplementary Information:**

The online version contains supplementary material available at 10.1186/s40104-023-00895-4.

## Background

Yeasts are single-celled members of the fungi kingdom, widely distributed in nature, capable of living in extreme environments. Its biological relevance is determined by 1,500 species described so far, with a high impact on both plant and animal life [1], being also a pillar in the human food industry. Used for millennia due to their fermentative activity to produce highly demanded foods (bread, cheese, wine, beer, among others), for some years now yeasts have also been in the crosshair of the pharmaceutical industry [2]. Some authors have suggested their use as transport vehicles for bioactive compounds [3], drug delivery [4], or what is more widely considered, as natural probiotics for humans, livestock, and aquaculture species [5, 6]. Some of them have been isolated from the intestine of several species as part of their commensal microbiota, including fish [7]. In this sense, the reasons why some yeast species are highly interesting for their use as probiotics is because they may promote somatic growth, modulate the intestinal microbiota, as well as enhance host health and condition due to their contents in immunomodators like β-glucans among other bioactive compounds [8–10].

Several studies have recommended the inclusion of yeasts in livestock and aquaculture feeds, as an additive, due to their beneficial effects in key performance indicators related to growth and feed efficiency, and in terms of antioxidant performance, and immunity enhancement, among which is the resistance to infections [11–13]. On the other hand, yeasts and their metabolic products from food industry by-products could also be used to produce high quality alternative protein sources within a sustainable and circular economy approach [14–16]. It is known that yeasts are a rich source of nutrients and protein-related compounds with a wide range of bioactive activities. In this sense, yeasts cells contain different types of wall-related compounds, like glucans and polyamines (biologically active amines), which are essential for the maintenance of life [17]. Specifically, amines have been observed to be relevant to the normal cell development and are involved in several cellular processes, along with systemic benefits for the animals [18–20].

Among all yeast species, *Saccharomyces cerevisiae* might be the most studied yeast as a probiotic or alternative protein source. However, another yeast species that has received a lot of attention for its probiotic effect is *Debaryomyces hansenii* [21]. Contrary to other species, *D. hansenii* belongs to the normal microbiota of wild and cultivated animals [22], and it has been reported that it also promotes growth and development in various species at different stages, results that have been attributed to its glucans and polyamine content [21]. This probiotic has been tested at early life stages in aquatic species with promising results; for instance, larvae of *Seriola rivoliana* [23], *Danio rerio* [24], *Dicentrarchus labrax* [25] and *Penaeus monodon* [26] showed improved survival and development when fed a diet supplemented with *D. hansenii.* Similarly, other studies focused on juveniles and adults showed that a diet supplemented with this yeast had a positive effect on the host in terms of its physiological and immune responses, showing its capacity to counteract possible stress impacts as well as to enhance disease resistance [17, 27, 28], along with an improvement of the intestinal health and function [29]. However, little is known about the effects on fish performance, and especially about the modes of action on the intestinal mucosa, beyond a purely immunological aspect.

The aim of this study was to evaluate the effect of a feed supplemented with *D. hansenii* on gilthead seabream (*Sparus aurata*) on common key performance indicators (KPI) associated to somatic growth and feed efficiency coupled with the analysis of the transcriptomic profile and the histological organization of the anterior-mid intestine, and its autochthonous microbiota in order to provide inside into the effects of this yeast on host condition. The convergence offered in this study by the integration of these analytics in the intestinal mucosa in one of the most highly valued species in the Mediterranean [30], is highly relevant for the introduction and management of sustainable feeding strategies for the growing aquaculture industry.

## Material and methods

### Diet composition

To evaluate the effects of *D. hansenii* as a probiotic in aquafeeds, two diets were formulated with similar levels of protein content (48.4% crude protein), lipid content (17.2% crude fat), and energy levels (21.7 MJ/kg gross energy) (Table 1). Both diets, named Control and Yeast diets, were formulated with low levels of fishmeal (7%) where fishmeal was replaced by a blend of plant-based meals (wheat, corn, pea and soy) (Table 1). Diets only differed in the inclusion of *D. hansenii* (CBS 8339) at an inclusion rate of 1.1% (17.2 × 10^5^ CFU). *D. hansenii* was provided by CIBNOR (La Paz, Mexico) and grown as described in Tovar-Ramírez et al. [25].

**Table 1:** Ingredients and proximal composition of the experimental diets

Ingredients, %	Diets	
	**Control**	**Yeast**
Fishmeal LT70^a^	7.0	7.0
Soy protein concentrate^b^	21.0	21.0
Pea protein concentrate	12.0	12.0
Wheat gluten	12.0	12.0
Corn gluten	12.0	12.0
Soybean meal 48	5.0	5.0
Wheat meal	10.4	10.4
Fish oil^c^	15.0	15.0
Vit & Min premix (PV01)^d^	1.0	1.0
Soy lecithin (Powder)	1.0	1.0
Binder (guar gum)	1.0	1.0
Monocalcium phosphate	2.0	2.0
*L*-Lysine	0.3	0.3
*L*-Tryptophan	0.1	0.1
*DL*-Methionine	0.2	0.2
Total, %	100	100
**Supplementation**		
Yeast (*Debaromyces hansenii*), %	-	1.1
**Feed basis proximate composition**		
Crude protein, % feed	48.4	48.4
Crude fat, % feed	17.2	17.2
Fiber, % feed	1.53	1.53
Ash, % feed	5.89	5.89
Gross energy, MJ/kg feed	21.7	21.7

^a^Fishmeal LT70, Norvik 70, Sopropêche, France

^b^SOYCOMIL®, ADM Animal Nutrition, Quincy, USA

^c^SAVINOR UTS, Trofa, Portugal

^d^Vitamin and mineral premix (PREMIX Sparos Lda, Portugal): Vitamins (IU or mg/kg diet): *DL*-alpha tocopherol acetate, 100 mg; sodium menadione bisulphate, 25 mg; retinyl acetate, 20,000 IU; *DL*-cholecalciferol, 2,000 IU; thiamin, 30 mg; riboflavin, 30 mg; pyridoxine, 20 mg; cyanocobalamin, 0.1 mg; n-icotinic acid, 200 mg; folic acid, 15 mg; ascorbic acid, 500 mg; inositol, 500 mg; biotin, 3 mg; calcium panthotenate, 100 mg; choline chloride, 1,000 mg, betaine, 500 mg. Minerals (g or mg/kg diet): copper sulfate, 9 mg; ferric sulfate, 6 mg; potassium iodide, 0.5 mg; manganese oxide, 9.6 mg; sodium selenite, 0.01 mg; zinc sulfate,7.5 mg; sodium chloride, 400 mg; excipient wheat middlings

Both diets (2 mm pellet size) were manufactured by SPAROS Lda (Olhão, Portugal) in a temperature-controlled low-shear extruder as detailed in Gisbert et al. [31]. Samples of each diet were taken for proximate composition, and feeds were stored at 4 °C along the experimental feeding trial to prevent their oxidation.

### Experimental model and design

A total of 500 *S. aurata* juveniles (body weight, BW = 14.5 ± 1.2 g; mean ± SD) were obtained from a commercial farm (PISCIMAR S.L.; AVRAMAR, Burriana, Spain). Fish were transported to IRTA-La Ràpita research facilities (La Ràpita, Spain), and acclimated for two weeks in two tanks of 2,000 L connected to a IRTAmar® recirculating system under natural photoperiod and constant temperature (23.0 ± 1.0 °C). During acclimatation, fish were fed with a commercial diet containing 48.5% protein, 18% lipid, and 18.5 MJ/kg digestible energy (Optibream, Skretting).

Before the trial, 200 fish (25 fish per tank; 4 tank replicates per diet) were gently anesthetized (50 mg/L tricaine methanesulfonate, MS-222, Sigma-Aldrich, Madrid, Spain) and individually measured in BW and standard length (SL) to the nearest 0.1 g and 1 mm, respectively. The trial lasted for 70 d, during which fish were fed at 3.5% of the stocked biomass by means of automatic feeders (ARVO-TEC T Drum 2000; Arvotec, Finland). The daily feed ratio was evenly distributed in 4 meals at 08:00, 11:00, 13:00 and 16:00 h (at each meal, the corresponding feed ration was distributed during 1 h). One hour after each meal, uneaten pellets were collected, dried overnight (120 °C), and weighted (g) for calculating daily feed ingesta values, while feed ration was adjusted to guarantee 10%–15% of uneaten pellets; thus, confirming that fish were fed at satiation. This trial was run in a recirculating system IRTAmar® under natural photoperiod conditions (14 h light/8 h dark), constant temperature (23.3 ± 1.3 °C), salinity (35–36 ppt), dissolved oxygen (5.7 ± 0.2 $\mathrm{mg}/\mathrm L$) (OXI330, Crison Instruments, Spain), and pH (8.2 ± 0.1) (pHmeter 507, Crison Instruments). Ammonia (0.13 ± 0.1 mg ${\mathrm{NH}}_{4}^{+}$/L) and nitrite levels (0.18 ± 0.1 mg ${\mathrm{NO}}_{2}^{-}$/L) (HACH DR 900 Colorimeter, Hach Company, Spain) were weekly controlled.

Growth and feed performance indicators were calculated using the following formulae:

- $$\mathrm{Specific}\;\mathrm{growth}\;\mathrm{rate}\;\mathrm{in}\;\mathrm{body}\;\mathrm{weight},\;\mathrm{BW}\;\left(\mathrm{SGR},\;\%\;\mathrm{BW}/\mathrm d\right)\:=\:100\;\times\;\left[\left(\ln\;{\mathrm{BW}}_{\mathrm f}\;-\;\ln\;{\mathrm{BW}}_{\mathrm i})\;/\;\mathrm d\right)\right];\;\mathrm{where}\;{\mathrm{BW}}_{\mathrm f}\;\mathrm{and}\;{\mathrm{BW}}_{\mathrm i}\;\mathrm{are}\;\mathrm{the}\;\mathrm{final}\;\mathrm{and}\;\mathrm{initial}\;\mathrm{mean}\;\mathrm{BW}\;\mathrm{of}\;\mathrm{fish}.$$  
- $$\mathrm{Survival}\;\mathrm{rate}\;(\mathrm{SR},\;\%)\:=\:{100\;\times\;(\mathrm{Final}\;\mathrm{number}\;\mathrm{of}\;\mathrm{fish}\;/\;\mathrm{Initial}\;\mathrm{number}\;\mathrm{of}\;\mathrm{fish}).}$$ 
- $$\mathrm{Feed}\;\mathrm{conversion}\;\mathrm{ratio}\;(\mathrm{FCR})\:=\:\mathrm{Feed}\;\mathrm{ingesta}\;(\mathrm g)\;/\;\mathrm{Increase}\;\mathrm{in}\;\mathrm{fish}\;\mathrm{biomass}\;(\mathrm g).$$ 

At the end of the trial, all fish were individually captured, anaesthetised (100 mg/L MS222) and their BW and SL measured as previously described. In addition, fifteen fish per tank were euthanised (300 mg/L MS222) and their digestive tract removed for further transcriptomic and microbiome studies as described below. In this sense, the anterior-mid intestine was chosen for transcriptomic purposes because of its immunological relevance in comparison with other intestinal sections [32, 33].

### Histological and histochemical analysis

Samples from the anterior-mid intestine (*n* = 12 randomly selected fish per experimental diet; 3 fish per tank) were embedded in paraffin and sagittally sectioned (5–6 μm). A total of 576 sections (2 per each sample × 24 samples × 12 histochemical techniques) were used for histological and histochemical purposes. Two sections per each sample were stained with haematoxylin–eosin for descriptive purposes; the rest were used for evaluating the histochemical properties of epithelial and mucous cells. In brief, Schiff, Periodic Acid Schiff (PAS), diastase-PAS and Alcian Blue (AB) pH 2.5, 1 and 0.5 (carboxylated and sulphated glycoconjugates/glycoproteins) techniques were used for studying carbohydrate distribution. Furthermore, several horseradish peroxidase (HRP) conjugated lectins (Sigma-Aldrich, Spain) were used for proper characterization of different glucidic residues bound to the glycoconjugates; in particular, *Canavalia ensiformes*/ConA (mannose and/or glucose), *Ulex europeus*/UEA-I (*L*-fucose), *Triticum vulgaris*/WGA (N-acetyl-*D*-glucosamine and/or N-acetyl-neuraminic acid, NeuNAc/sialic acid/NANA), *Glycine max*/SBA (a-N-acetyl-*D*-galactosamine) and *Sambucus nigra*/SNA (NeuNAc/sialic acid/NANA). Lectin concentrations ranged between 15 μg/L to 30 μg/mL. Regarding negative controls, omission of the respective lectin, substitution of lectin-HPR conjugates by TBS and treatments with different enzymes were performed according to Sarasquete et al. [34]. The peroxidase activity was visualized with 3,3-diaminobenzidine tetra hydrochloride/DAB and hydrogen peroxide (0.05%). All the techniques were performed according to Underwood [35] and following proper standardized techniques and protocols [36]. All reagents were purchased from Sigma-Aldrich (St Louis, MO, USA).

Histological images (600 dpi) were taken with a Leitz Wetzlar microscope with a built-in SPOT Insight Color camera (Ernst Leitz Wetzlar GmbH, Germany). Results were manually registered using a semi-quantitative assessment scoring based on color intensity scores (0, negative; 1, weak; 2, moderate; 3, intense; 4, very intense) from four independent observers, comparing the sections of the control with the experimental diet. The mucous cell count was determined in four different sites of each histological section, and the number of cells expressed per length unit of the basal lamina of the mucosal epithelium (1 mm) according to Yamamoto et al. [37].

### Intestinal microbiota

#### DNA extraction and sequencing by Illumina MiSeq technology

The complete intestine of *S. aurata* (*n* = 8 per dietary condition) was taken for microbiome analyses. Samples were stored at −20 °C, thawed gradually on ice and the mucus intestinal contents (autochthonous microbiota) were collected for further analyses. A 1-mL aliquot per sample of intestinal contents were collected with 1 mL PBS. Total DNA was extracted from each sample according to protocol described in Tapia-Paniagua et al. [38], with minor modifications. DNA concentration and purity were quantified fluorometrically with Qubit™ dsDNA HS Assay Kit (Thermo Fisher Scientific, Waltham, MA, USA) and by spectrophotometric and electrophoretic methods to study the degree of purity, quality, and DNA integrity. Isolated DNA was stored at –20 ºC until further processing and 30 ng used for subsequent analyses. 16S rRNA of samples was sequenced on Illumina MiSeq platform (Illumina, San Diego, CA, USA) with 2 × 300 bp paired-end sequencing in the Ultrasequencing Service of Malaga (Malaga, Spain). Sequencing was carried out using the primers 341F and 805R directed to the variable regions V3–V4 of the 16S rRNA gene [39]. Raw read sequences of the 16S rRNA gene from *S. aurata* intestinal microbiota are publicly available in the NCBI SRA depository (SRS16609731—SRS16609735) within BioProject PRJNA914904, with BioSample accession numbers SAMN32318396-SAMN32318408.

#### Bioinformatic analysis

Primers were removed and quality control were performed with *cutadapt* and *FastQC* software respectively. Barcoding sequences were processed using R *DADA2* library. Briefly, forward and reverse reads were truncated with decreasing quality metrics while maintaining sequence overlap. Paired reads were assembled after error modelling and correction, creating amplicon sequence variants (ASVs). Chimeric ASVs were removed by reconstruction against more abundant parent ASVs. Taxonomy was assigned to representative sequence variants using SILVA release 138 database, clustered at 99% identity, and trimmed to the amplified region. Abundance of ASV of the intestinal microbiota was processed using *phyloseq* and *vegan* library in R statistical package. Shannon's alpha diversity indices, to evaluate taxonomic diversity and PCoA to beta diversity were performed. ASVs with abundance minor to 10 reads in at least of 10% of samples were filtered to taxonomy analyses. R library *ggplot* was used to represent the abundance of different taxonomy categories and differential abundance of taxa was carried out using the R package DESeq2 with a false discovery rate (FDR < 0.05).

### Gut transcriptional analysis

#### RNA isolation and quality control

Total RNA from the anterior-mid intestine of nine randomly selected fish per dietary treatment (*n* = 4 fish per tank) was extracted using the RNeasy® Mini Kit (Qiagen, Germany). Total RNA was eluted in a final volume of 35 μL nuclease-free water and treated with DNAse (DNA-free™ DNA Removal Kit; Invitrogen, Lithuania). Total RNA concentration and purity were measured using Nanodrop-2000® spectrophotometer (Thermo Scientific, USA) and stored at −80 °C until analysis. Prior to hybridization with microarrays, RNA samples were diluted to 133.33 ng/μL concentration, checked for RNA integrity (Agilent 2100 Bioanalyzer; Agilent Technologies, Spain) and selected by the criteria of a RIN value > 8.5. Three different pools of samples per dietary treatment were established (*n* = 4 fish each pool); this choice of pooling individual samples resulted in the loss of information derived from individual sample variability.

#### Microarray hybridization and analysis

Anterior-mid intestine transcriptional analysis from both experimental groups was carried out using the Aquagenomics *Sparus aurata* Oligonucleotide Microarray v2.0 (4 × 44 K) (SAQ) platform. Detailed information and transcriptomic raw data are available at the Gene Expression Omnibus (GEO) public repository at the US National Center for Biotechnology Information (NCBI), accession numbers GPL13442, and GSE162504, respectively. Sample labelling, hybridization, washes, and scanning was performed as described in Firmino et al. [40]. Briefly, a one-color RNA labelling was used (Agilent One-Color RNA Spike-In kit; Agilent Technologies, USA). RNA from each sample pool (200 ng) was reverse-transcribed with spike-in. Then, total RNA was used as template for Cyanine-3 (Cy3) labelled cRNA synthesis and amplified with the Quick Amp Labelling kit (Agilent Technologies). cRNA samples were purified using the RNeasy® micro kit (Qiagen). Dye incorporation and cRNA yield were checked (NanoDrop ND-2000® spectrophotometer). Then, Cy3-labeled cRNA (1.5 mg) with specific activity > 6.0 pmol Cy3/mg cRNA was fragmented at 60 °C for 30 min and hybridized with the array in presence of hybridization buffer (Gene expression hybridization kit, Agilent Technologies) at 65 °C for 17 h. For washes, microarrays were incubated with Gene expression wash buffers, and stabilization and drying solution according to manufacturer instructions (Agilent Technologies). Microarray slides were then scanned (Agilent G2505B Microarray Scanner System), and spot intensities and other quality control features extracted (Agilent Feature Extraction software version 10.4.0.0). The extracted raw data were imported and analysed with GeneSpring (version 14.5 GX software, Agilent Technologies). To standardize the arrays intensity, 75% percentile normalization was used, and data were filtered by expression levels. The differential expressed genes (DEGs) were obtained from a gene-level differential expression analysis. Expression values with up-value < 0.05 were considered statistically significant. The DEGs were grouped according to their fold-change value (*P*-value < 0.05) and represented using the GraphPad softwarev7.0 for Windows. The 3D principal component analysis (PCA) was carried out using GeneSpring software (Agilent), four eigenvectors were calculated to describe the aggrupation of diets control and yeast in a 3D plot. The gene expression values (log-expression ratios) were represented by a hierarchical clustering heatmap analysis using GeneSpring software (Agilent).

The Search Tool for the Retrieval of Interacting Genes (STRING) public repository version 11.0 (https://string-db.org), and the application ClueGO (v2.5.9) and CluePedia (v1.5.9) using Cytoscape (v3.9.1) software, were used to generate the anterior-mid intestine transcripteractome and cluster aggrupation for the DEGs of fish fed the yeast-supplemented diet. A protein–protein interaction (PPI) Networks Functional Enrichment Analysis for all the differentially expressed genes (DEGs) was conducted with a medium-confidence interaction score (0.4) using Homo sapiens as model organism [40]. Gene Ontology (GO) and Kyoto Encyclopedia of Genes and Genomes (KEGG) enrichment analysis of all the DEGs obtained were also assessed through STRING (*P* < 0.05). In order to confirm match of gene acronyms between both *Homo sapiens* and *S. aurata* species, human orthology identification based on gene/protein name was accessed through the Genecards (www.genecards.org) [41] and Uniprot (www.uniprot.org) databases. Additionally, protein–protein BLAST (BLASTp) was run (E < 10^−7^; query cover > 95%).

### Statistical analysis

Data on growth, feed performance indicators and goblet cell density in the intestinal epithelium are expressed as mean ± standard deviation, and a probability value of *P* < 0.05 was considered as significant by means of a Student’s *t*-test. Regarding microbiota studies, F diversity indexes were compared between both dietary groups by means of a Student’s *t*-test (*P* < 0.05).

## Results

### Growth and feed performance indicators

At the end of the trial, fish fed low fish meal diet supplemented with *D. hansenii* (1.1%, 17.2 × 10^5^ CFU; Yeast diet) had significant differences compared with the fish fed the Control diet in terms of somatic growth. Data showed improved BW_f_, BWG and the SGR values in fish fed the Yeast diet compared with those fed the Control diet (Table 2; *P* < 0.05). Furthermore, FCR values were significantly lower in fish fed the Yeast diet when compared to the control ones (1.26 ± 0.07 vs. 1.06 ± 0.05, respectively). No significant differences in the FI and SR were found between both groups (Table 2; *P* > 0.05).

**Table 2:** Growth and feed performance indicators in gilthead sea bream (*Sparus aurata*) fed experimental low fishmeal diets containing *Debaryomyces hansenii* (1.1%, 17.2 × 10^5^ CFU; Yeast diet) or devoid of the probiotic (Control diet)

	**Control diet**	**Yeast diet**	***P*-value**
Final body weight (BW_f_), g	84.4 ± 2.92	95.6 ± 4.64	0.007^*^
Body weight gain (BWG), g	69.9 ± 2.92	81.1 ± 4.65	0.007^*^
Specific growth rate (SGR), %BW/d	2.52 ± 0.05	2.69 ± 0.07	0.006^*^
Survival rate (SR), %	92.0 ± 3.3	93.0 ± 2.0	0.620
Feed intake (FI), g/fish	86.9 ± 3.77	84.3 ± 6.65	0.526
Feed conversion ratio (FCR), BWG/FI	1.26 ± 0.07	1.06 ± 0.05	0.003^*^

Data are shown as the mean ± standard deviation (SD). The asterisk (*) denotes statistically significant differences between both groups (Student’s *t-*test,* P* < 0.05; *n* = 4)

### Morphological organization of the anterior-mid intestine and histochemical properties of mucins produced by goblet cells

**Fig. 1:**
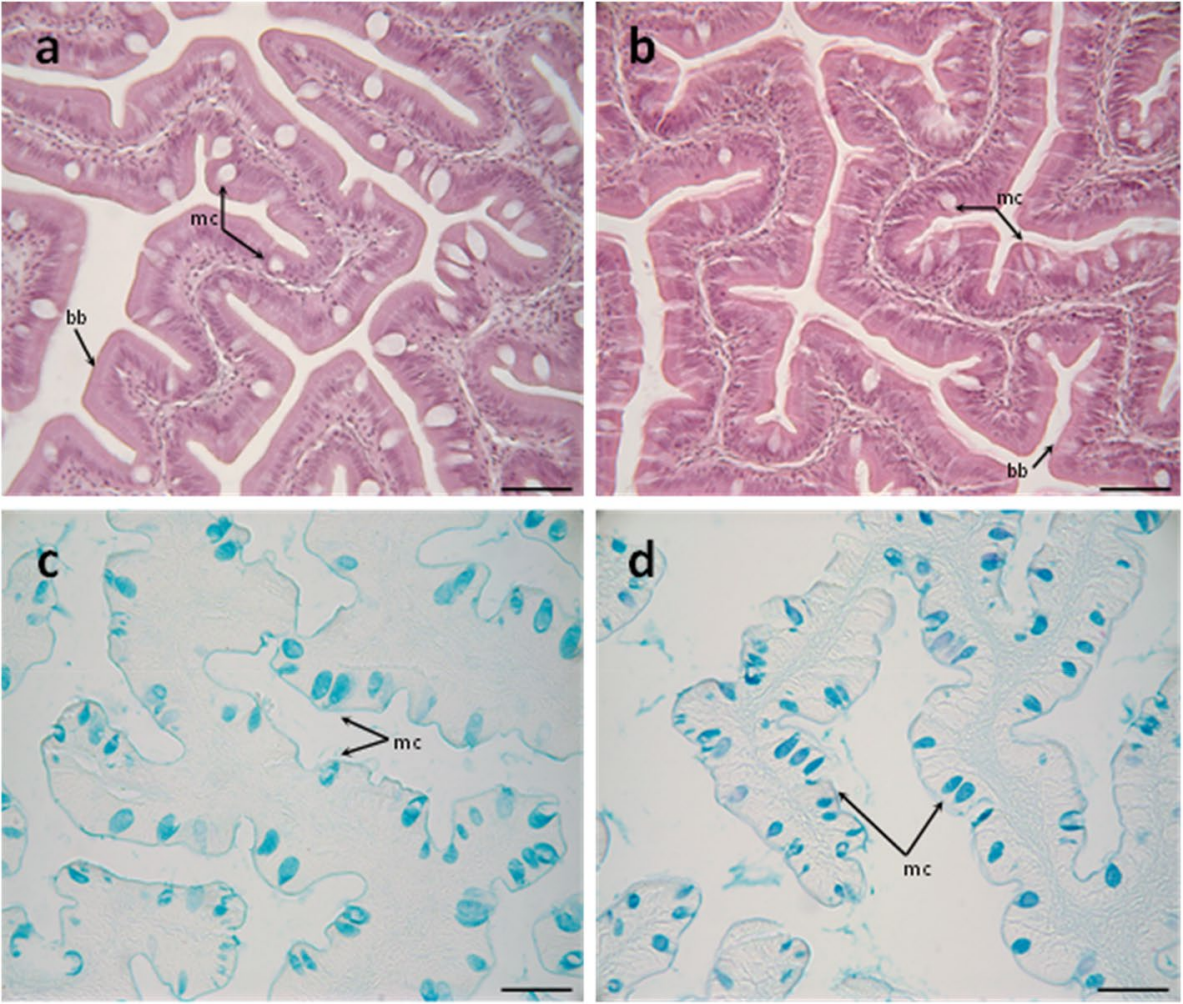
Histological organization of the anterior-mid intestine of gilthead seabream (*Sparus aurata*) fed experimental low fishmeal diets containing *Debaryomyces hansenii* (1.1%, 17.2 × 10^5^ CFU; Yeast diet) (**a**) or devoid of the probiotic (Control diet) (**b**). Histochemical properties of mucins produced by intestinal goblet cells regarding their content on carboxylated and/or sulphated acidic groups from fish fed the Control diet (**c**) and the Yeast diet (**d**). Abbreviations: mc, mucous cells; bb, brush border. Staining: hematoxylin–eosin (**a**, **b**), Alcian Blue pH = 2.5 (**c**, **d**). Scale bar = 50 µm

**Fig. 2:**
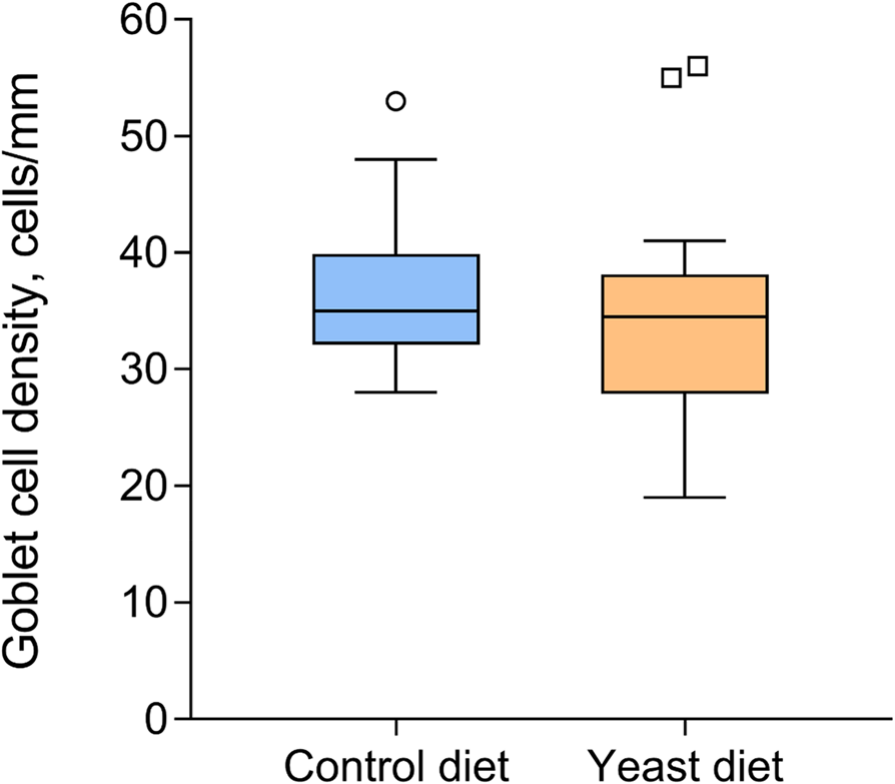
Box-plot graph of goblet cell density in the anterior-mid intestine of gilthead seabream (*Sparus aurata*) fed experimental low fishmeal diets containing *Debaryomyces hansenii* (1.1%, 17.2 × 10^5^ CFU; Yeast diet) or devoid of the probiotic (Control diet). No statistically significant differences were detected among dietary treatments (Student’s *t*-test; *P* > 0.05). Mean line within the boxplot indicate the average value of the series; O and ◻ = outlier points of the series

The mucosal layers from the anterior-mid intestine of fish fed both experimental diets were similar. In brief, both were lined by a simple columnar epithelium with basal nuclei, basophilic cytoplasm, and prominent brush border, with scattered goblet cells. No signals of histological alterations associated with inflammatory processes nor lymphocyte infiltrations in the anterior-mid intestine were observed in fish fed the Yeast diet compared to the control group (Fig. 1a and b). No significant differences in goblet cell density were detected when comparing both treatments (Fig. 2).

**Fig. 3:**
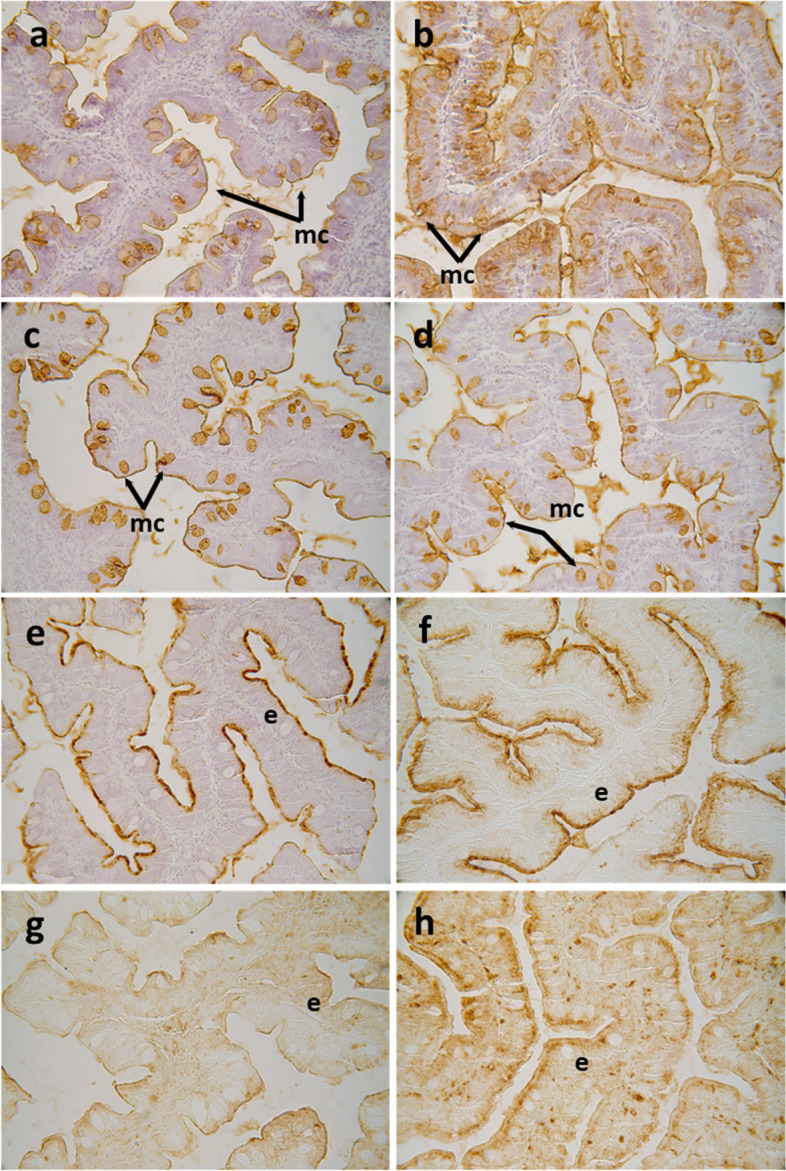
Histochemical localization of glycoconjugates containing sugar residues in the anterior-mid intestine of gilthead seabream (*Sparus aurata*) fed experimental low fishmeal diets containing *Debaryomyces hansenii* (1.1%, 17.2 × 10^5^ CFU; Yeast diet) or devoid of the probiotic (Control diet). Presence of glycoconjugates containing α/β-N-acetyl-*D*-galactosamine in mucous cells of *S. aurata* fed a Control (**a**) or a Yeast diet (**b**). Results denote a moderate increase in affinity for the SBA lectin in the mucous cells and in the intestinal epithelium from Yeast diet. Glycoconjugates containing N-acetyl-*D*-glucosamine and/or N-acetylneuraminic acid residues in mucous cells of *S. aurata* fed the Control or the Yeast diet (**d**). Results denote a moderate decrease in affinity for the WGA lectin in the mucous cells from the Yeast diet. Histochemical detection of glycoconjugates containing N-acetylneuraminic acid/sialic acid residues in the intestine from the Control (**e**) or Yeast (**f**) groups. Note the decrease in affinity for the SNA lectin in the anterior-mid intestinal epithelium of *S. aurata* fed the Yeast diet. Glycoconjugates containing a-mannose/a-glucose residues in anterior-mid intestine from *S. aurata* fed Control (**g**) or Yeast diets (**h**). Observe the increase in affinity for the ConA lectin in the intestinal epithelium of *S. aurata* fed the Diet Y. Abbreviations: e: epithelium; mc: mucous cells. Scale bar = 50 μm

Regarding the histochemical properties of mucins produced by goblet cells from the anterior-mid intestine, results showed a variable richness of neutral glycoproteins (PAS and diastase-PAS positive) (Table 3). In addition, mucins showed a mixture of carboxylated (AB pH = 2.5) and sulphated acidic groups (weak and strongly ionized; AB pH = 1.0 and 0.5, respectively) (Table 3 and Fig. 1c). Regarding lectin histochemistry, a specific affinity for WGA, SNA, ConA and SBA lectins was detected in the mucinous content of goblet cells (Table 3 and Fig. 3). Moreover, no variations were detected in the distribution of mucosal cell glycoconjugates between the upper and the bottom areas of the intestinal folds. When comparing both dietary groups, the dietary administration of *D. hansenii* modified the composition of glycoproteins of mucins produced by goblet cells, with an increase in the staining intensity of mucins rich in carboxylated and weakly sulphated glycoconjugates (Table 3 and Fig. 1c and d). In addition, an increase in affinity for SBA lectin and a decrease in the affinity for the WGA lectin was found in the mucinous content of goblet cells, whereas no changes were detected regarding ConA, SNA and UEA-I lectins (Table 3 and Fig. 3).

**Table 3:** Histochemical results and affinity for different lectins in the anterior-mid intestinal mucosa of gilthead seabream (*Sparus aurata*) fed experimental low fishmeal diets containing *Debaryomyces hansenii* (1.1%, 17.2 × 10^5^ CFU; Yeast diet) or devoid of the probiotic (Control diet)

	**Control diet**	**Yeast diet**
**General histochemistry**		
Neutral glycoproteins	1–2	1–2
Carboxylated glycoproteins	1–3	1–4
Weakly inonised sulphated glycoconjugates	2–3	3
Strongly ionised sulphated glycoconjugates	2–3	2–3
**Lectin histochemistry**		
ConA (Man/Glu)	0–1	0–1
WGA (βGlcNAc > NeuNAc/sialic acids/NANA)	2–3	1–2
SNA (Neur5Acα2; sialic acids/NANA)	0–1	0–1
SBA (α/β GalNAc)	0–3	2–3
UEA-I (*L*-Fuc)	0	0

Semi-quantitative assessment scoring based on color intensity scores: 0, negative (non-detected); 1, weak; 2, moderate; 3, intense; 4, very intense

### Intestinal microbiota

#### Sequencing data analysis

After bioinformatic processing, an average of 117,038.2 reads per sample were obtained (minimum reading 71,378 and maximum 162,602 reading) that were classified into 1,594 ASVs.

The Shannon and Simpson alpha diversity indexes were calculated for each sample of experimental groups (Table 4). The alfa diversity values in the experimental groups did not show significant differences (*P* > 0.05). The PCoA analysis to check the beta diversity between the detected sequences did not show a clearly separated grouping of samples based on the diet (Additional file 1: Fig. S1).

**Table 4:** Alpha diversity indices of microbiota intestine population of gilthead seabream (*Sparus aurata*) fed experimental low fishmeal diets containing *Debaryomyces hansenii* (1.1%, 17.2 × 10^5^ CFU; Yeast diet) or devoid of the probiotic (Control diet)

	**Observed**	**Shannon**	**Simpson**	**Chao1**	**ACE**
Control diet	206.6 ± 46.6	3.77 ± 0.19	0.94 ± 0.03	216.4 ± 38.5	216.4 ± 38.5
Yeast diet	203.2 ± 51.5	3.83 ± 0.38	0,95 ± 0.03	203.2 ± 38.5	203.2 ± 51.5

Mean ± standard deviation. No significant differences were found (Student’s *t*-test, *P* > 0.05)

#### Taxonomic composition of the intestinal microbiota

**Fig. 4:**
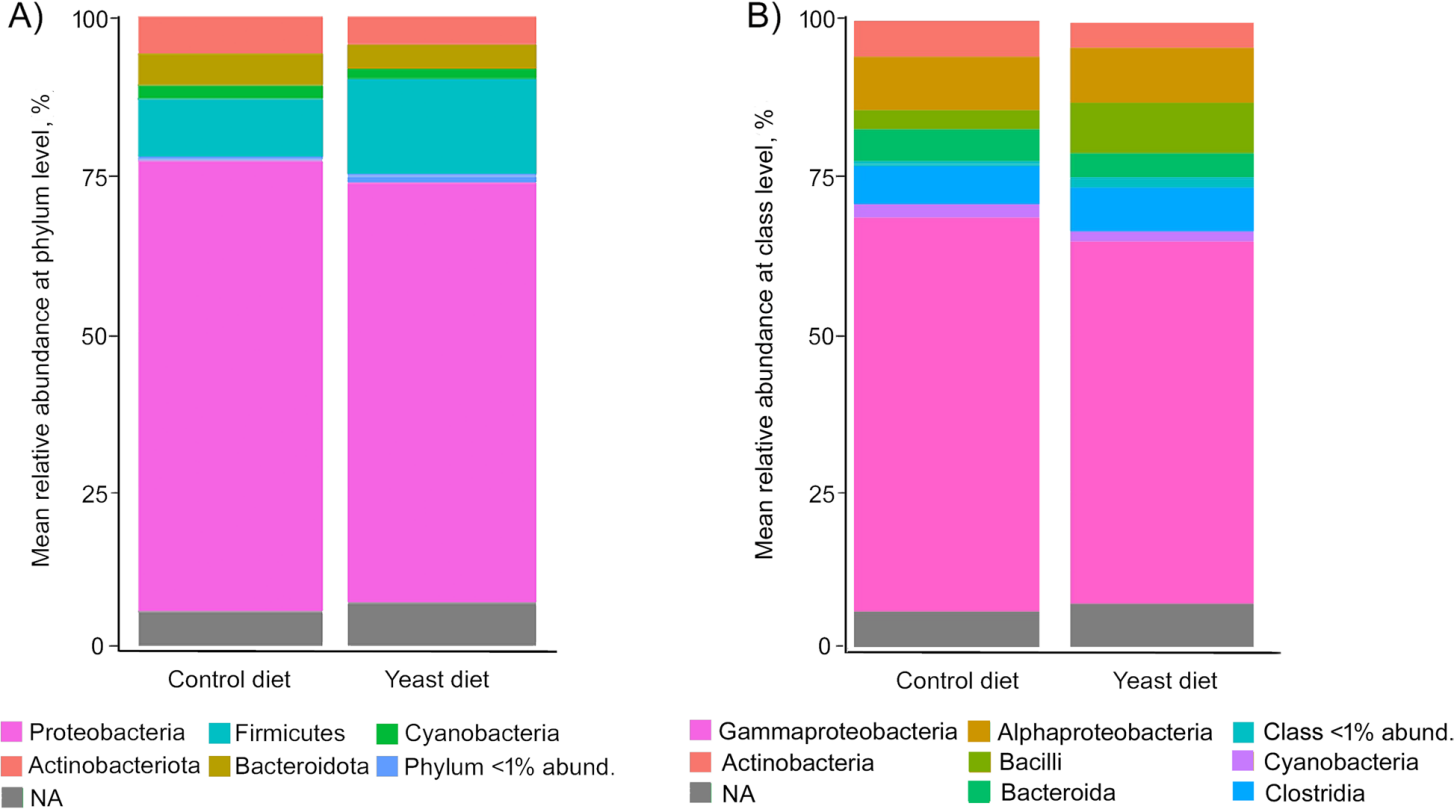
Values of relative abundance at the phylum (**A**) and class (**B**) level, obtained for the anterior-mid intestine microbiota of gilthead seabream (*Sparus aurata*) fed experimental low fishmeal diets containing *Debaryomyces hansenii* (1.1%, 17.2 × 10^5^ CFU; Yeast diet) or devoid of the probiotic (Control diet)

**Fig. 5:**
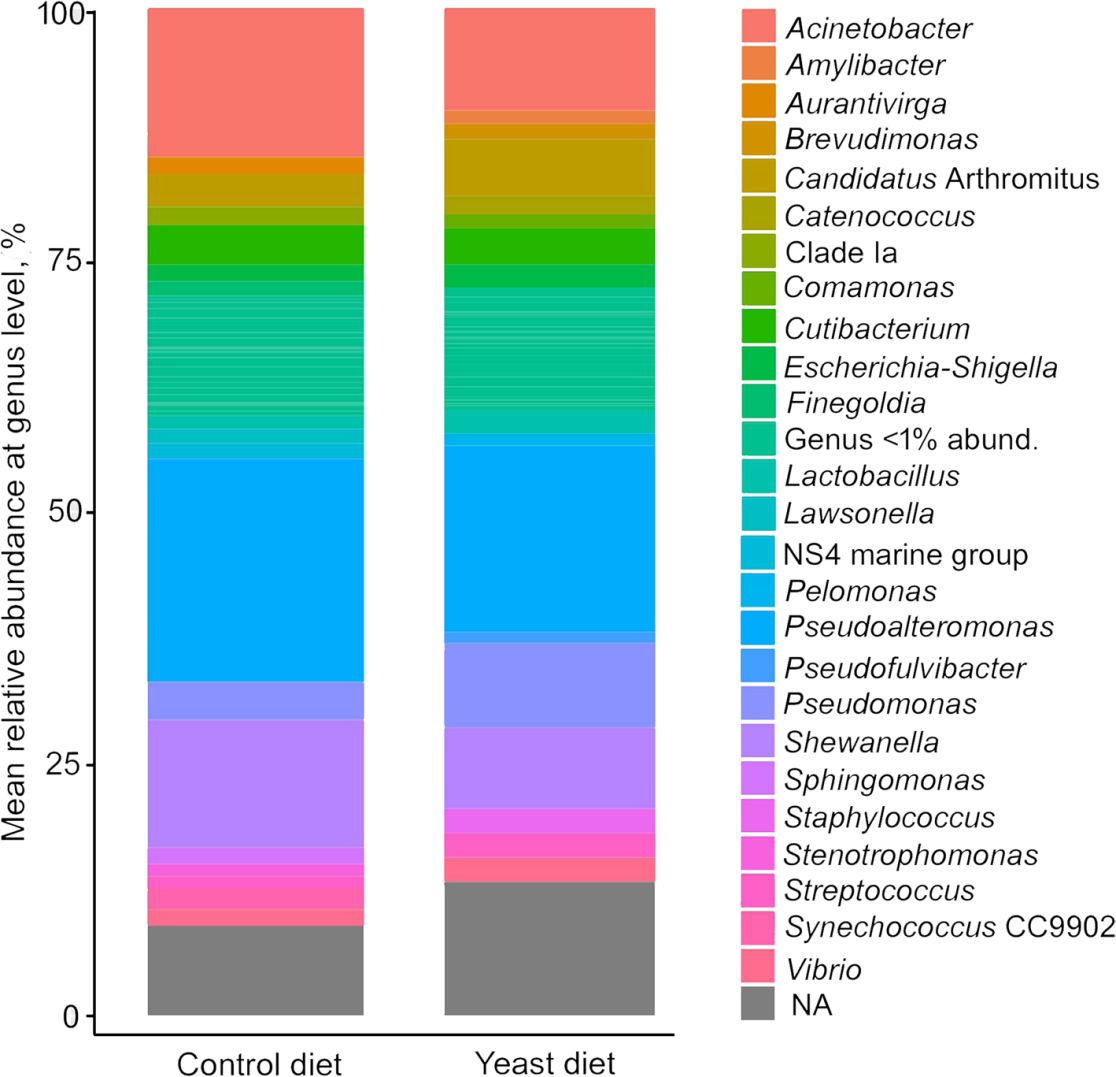
Values of relative abundance at genus level, obtained for the anterior-mid intestine microbiota of gilthead seabream (*Sparus aurata*) fed experimental low fishmeal diets containing *Debaryomyces hansenii* (1.1%, 17.2 × 10^5^ CFU; Yeast diet) or devoid of the probiotic (Control diet)

At phylum level, the predominant phyla detected in all fish fed both experimental diets were Proteobacteria, followed by Firmicutes (Fig. 4A), whereas the most abundant classes in all cases were alpha- and gamma-*Proteobacteria* (Fig. 4B). However, no significant differences were found among diets. A comparative analysis at genus level (abundance > 0.5%) showed a very similar abundance of sequences between specimens from both diets (Fig. 5), whereas *Pseudoalteromonas*, *Acinetobacter*, *Pseudomonas*, *Shewanella* and *Vibrio* were the most abundant genera.

**Fig. 6:**
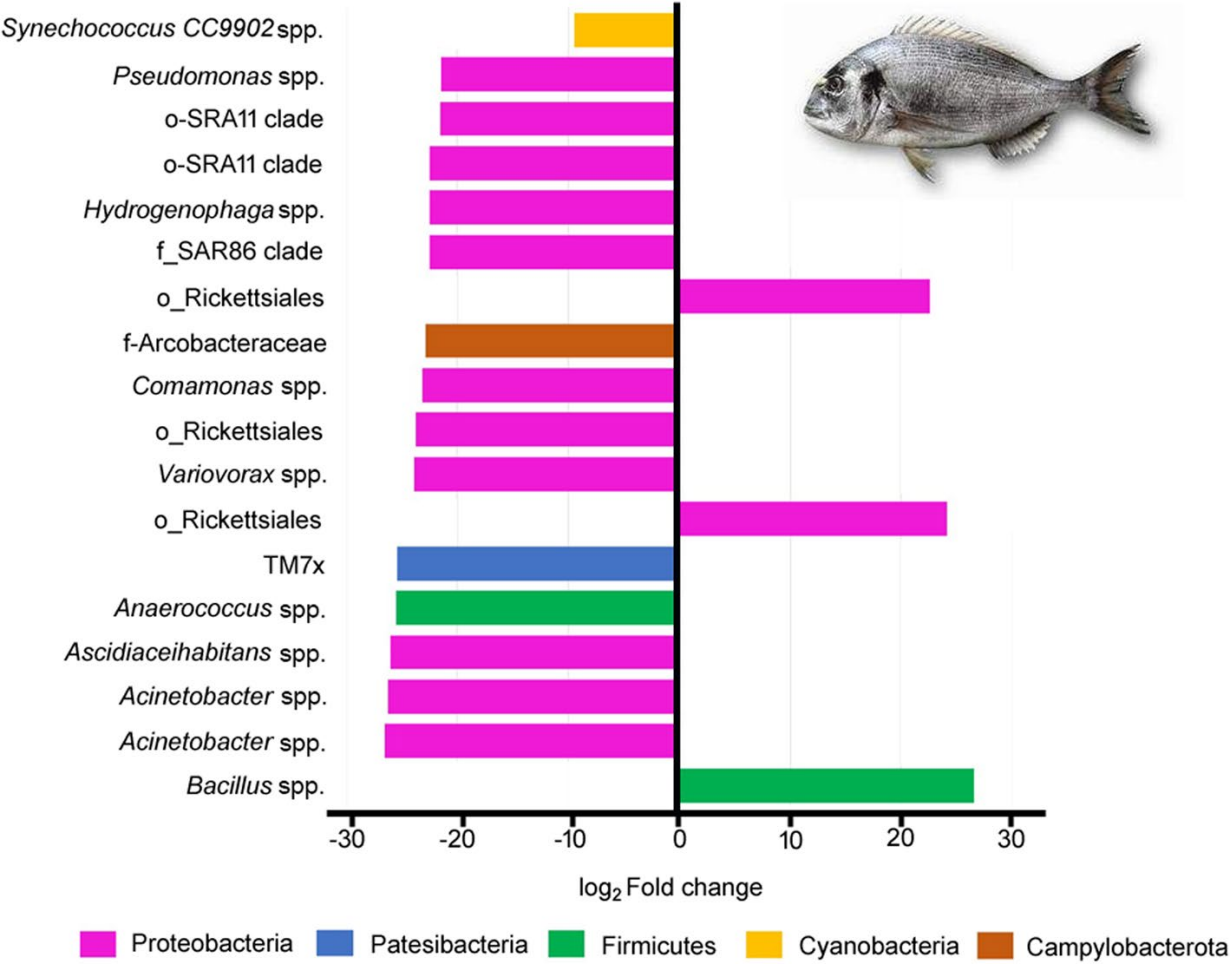
Logarithmic fold change of differentially abundant ASVs (DESeq2, FDR < 0.05) at the genus level in the anterior-mid intestine microbiota of gilthead seabream (*Sparus aurata*) fed experimental low fishmeal diets containing *Debaryomyces hansenii* (1.1%, 17.2 × 10^5^ CFU; Yeast diet) or devoid of the probiotic (Control diet)

DESeq2 analysis was applied to evaluate the significant differences (FDR < 0.05) between the abundance of the microbial taxa. Significant differences were detected regarding to the genera included in Protobacteria, and with abundance higher than 0.5% such as *Acinetobacter, Comamonas* and *Pseudomonas*, which showed decreased abundances in fish from the Yeast diet in comparison to those fed the Control diet (Fig. 6). Other significant differences were also detected within the Proteobacteria phylum, and particular in bacteria from the genus *Anaerococcus, Ascidiaceihabitans, Hydrogenophaga* and *Variovorax* that displayed lower abundances of ASVs in fish from the Yeast diet, whereas an increase of ASVs related to the genus *Bacillus* were found in the intestine of fish fed the Yeast diet (Fig. 6).

### Transcriptomics of the anterior-mid intestine

#### Transcriptomic profile

**Fig. 7:**
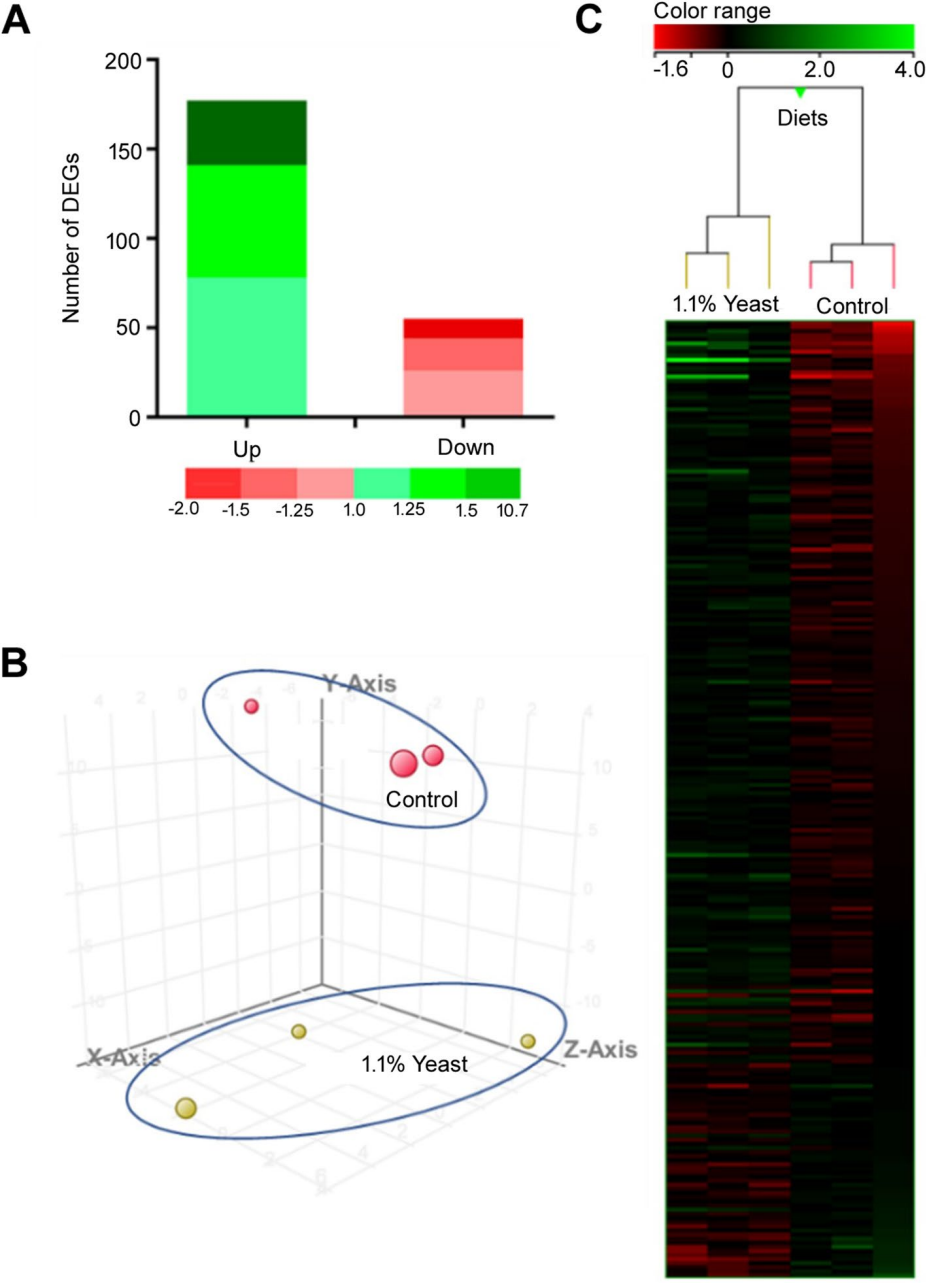
Transcriptomic response of the anterior-mid intestine of gilthead seabream (*Sparus aurata*) fed experimental low fishmeal diets containing *Debaryomyces hansenii* (1.1%, 17.2 × 10^5^ CFU; Yeast diet) or devoid of the probiotic (Control diet). **A** The green (up-regulated) and red (down-regulated) colour scheme indicate the gene modulation according to its magnitude interval (fold-change). **B** 3D principal component analysis (PCA). Control (red spheres) and *D. hansenii* groups (yellow spheres) are represented (each array included in the study is represented by one sphere). **C** Hierarchical clustering heatmap representing the 232 DEGs. The results of each microarray analysed for the control and yeast groups are shown. The green (up-regulation) and red (down-regulation) colour scheme indicates the gene normalized intensity values according to its magnitude interval

To evaluate the modulatory effects of the dietary administration of *D. hansenii* at 1.1% upon intestine transcriptome, we performed a microarray-based transcriptomic analysis. As shown in Fig. 7 and listed in Additional file 1: Table S1, a total of 232 differentially-expressed genes (DEGs) were found in the anterior-mid intestine when comparing samples from both diets (*P* < 0.05). Among these, 177 DEGs were up-regulated, and 55 DEGs were down-regulated showing both different levels of fold-change (FC) (Fig. 7A). The percentage expression pattern was similar in both up- and down-regulated genes, although in numerical terms most of the DEGs found were up-regulated with a FC > 2 (12 DEGs up-regulated vs. 1 DEG down-regulated), even some of these being expressed with a FC of 4, 6 or 10 (Fig. 7A and Additional file 1: Table S1). The 3D PCA analysis showed a clear grouping for both the Control and Yeast diets (Fig. 7B), meanwhile the hierarchical clustering heatmap confirmed the conservative response of the arrays, showing a clear differential expression profile of DEGs between both dietary groups (Fig. 7C).

**Fig. 8:**
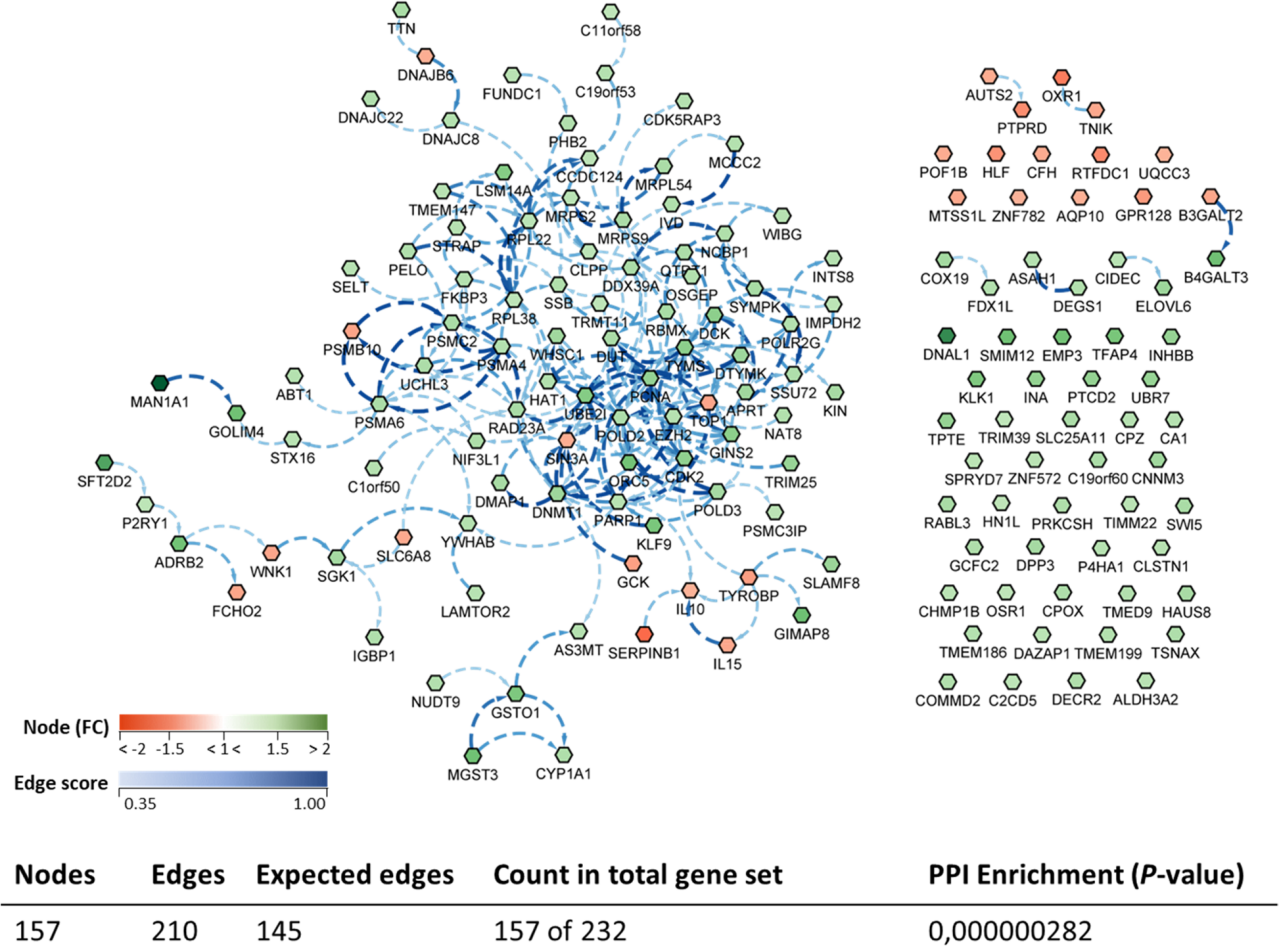
Biological network of the differentially expressed genes (DEGs) in the anterior-mid intestine of gilthead seabream (*Sparus aurata*) fed experimental low fishmeal diets containing *Debaryomyces hansenii* (1.1%, 17.2 × 10^5^ CFU; Yeast diet) or devoid of the probiotic (Control diet). Node fill with a continuous mapping colour from red (down-regulated DEGs) to green (up-regulated DEGs), represents the fold change (FC) intensity. Edge score was represented by doted lines with a continuous mapping from 0.35 (light Blue) to 1.00 (dark blue). The transcripteractome was obtained using Cytoscape (v3.9.1) platform with String (v1.7.1). For details, please refer to Additional file 1: Table S1

A biological network (transcripteractome) considering the complete DEGs annotated list was prepared in order to have an overview of the relationships between them (Fig. 8). The gene network resulted in 157 nodes/DEGs and 210 edges/interactions (with an expected number of 145 edges), excluding from the analysis the remaining DEGs (*n* = 75; 32% of the total DEGs), classified as unknown genes.

#### Functional enrichment analysis

The functional enrichment analysis of the transcripteractome helped us to identify several biological processes and pathways for the DEGs in the anterior-mid intestine of *S. aurata* fed the Yeast diet in comparison to their congeners from the control group (Control diet). Thus, 54 GO were identified (*P* < 0.05; Additional file 1: Table S2), whereas the top-10 representative processes per number of DEGs were as listed: “Nitrogen compound metabolic process” (60.5% of the total DEGs: 83 up-regulated vs. 12 down-regulated; GO:0006807), “Organic substance biosynthetic process” (30.6% of the total DEGs: 42 up-regulated vs. 6 down-regulated; GO:1901576), “Cellular response to chemical stimulus” (28.2% of the total DEGs: 35 up regulated vs. 9 down-regulated; GO:0070887), “Nucleic acid metabolic process” (24.8% of the total DEGs: 37 up-regulated vs. 2 down-regulated; GO:0090304), “Negative regulation of gene expression” (20.4% of the total DEGs: 27 up-regulated vs. 5 down-regulated; GO:0010629), “Cellular catabolic process” (18.5% of the total DEGs: 28 up-regulated vs. 1 down-regulated; GO:0044248), “Heterocycle biosynthetic process” (14.0% of the total DEGs: 21 up-regulated vs. 1 down-regulated; GO:0,018130), “Cellular response to oxygen-containing compound” (13.4% of the total DEGs: 16 up-regulated vs. 5 down-regulated; GO:1901701), “DNA metabolic process” (12.1% of the total DEGs: 17 up-regulated vs. 2 down-regulated; GO:0006259) and, “Symbiotic process” (11.5% of the total DEGs: 15 up-regulated vs. 3 down-regulated; GO:0044403) (Additional file 1: Table S3).

**Fig. 9:**
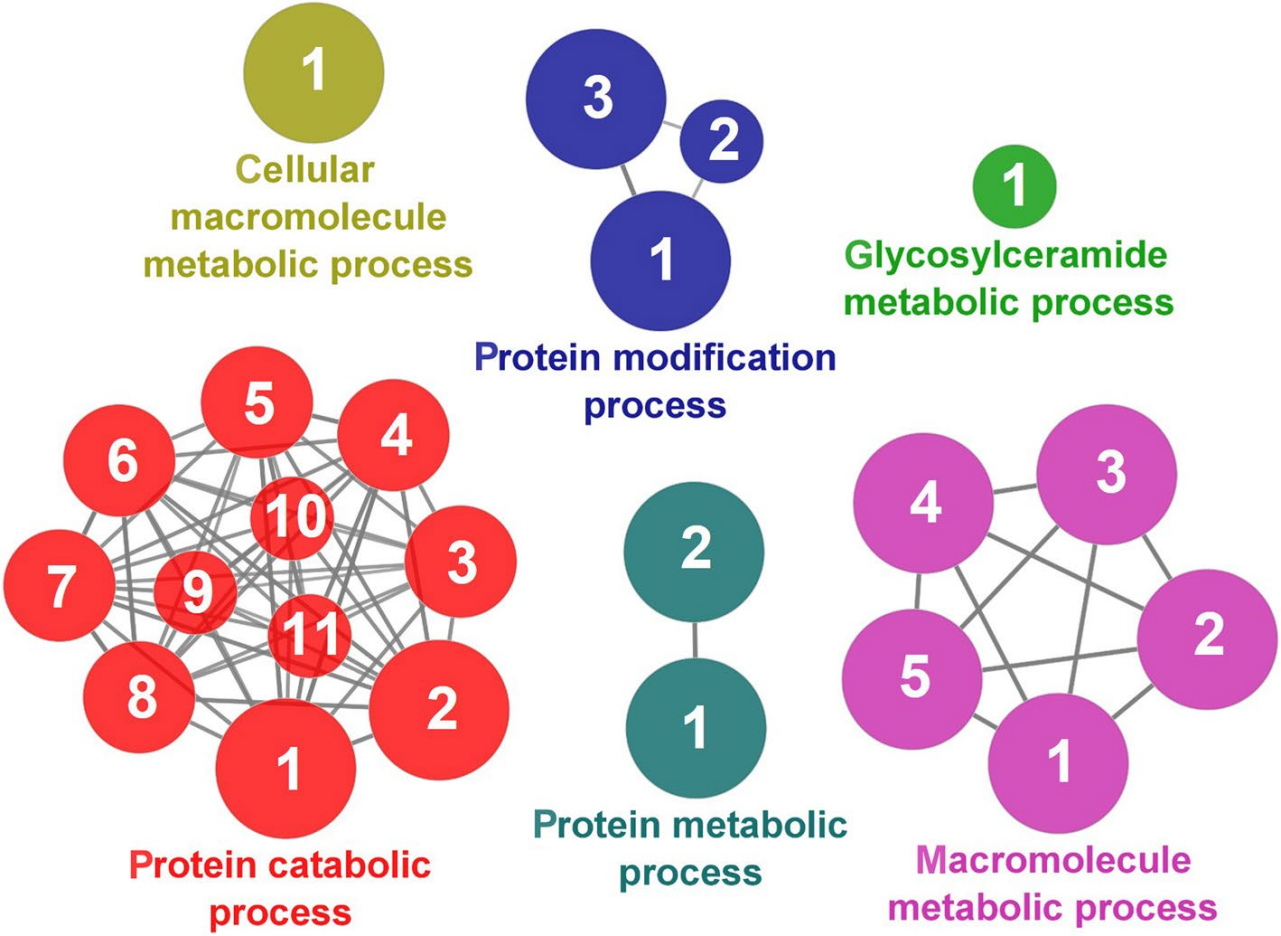
Protein metabolism cluster of the functional enrichment network for biological processes based on the related functions of the differentially expressed genes (DEGs) in the anterior-mid intestine of gilthead seabream (*Sparus aurata*) fed experimental low fishmeal diets containing *Debaryomyces hansenii* (1.1%, 17.2 × 10^5^ CFU; Yeast diet) or devoid of the probiotic (Control diet). The node size determinate the significance (Bonferroni step-down correction; small *P* < 0.05; medium *P* < 0.005; large *P* < 0.0005), the node colour determinate the principal GO process aggrupation, and the node numbers indicate different GO process classified in Additional file 1: Table S4

**Fig. 10:**
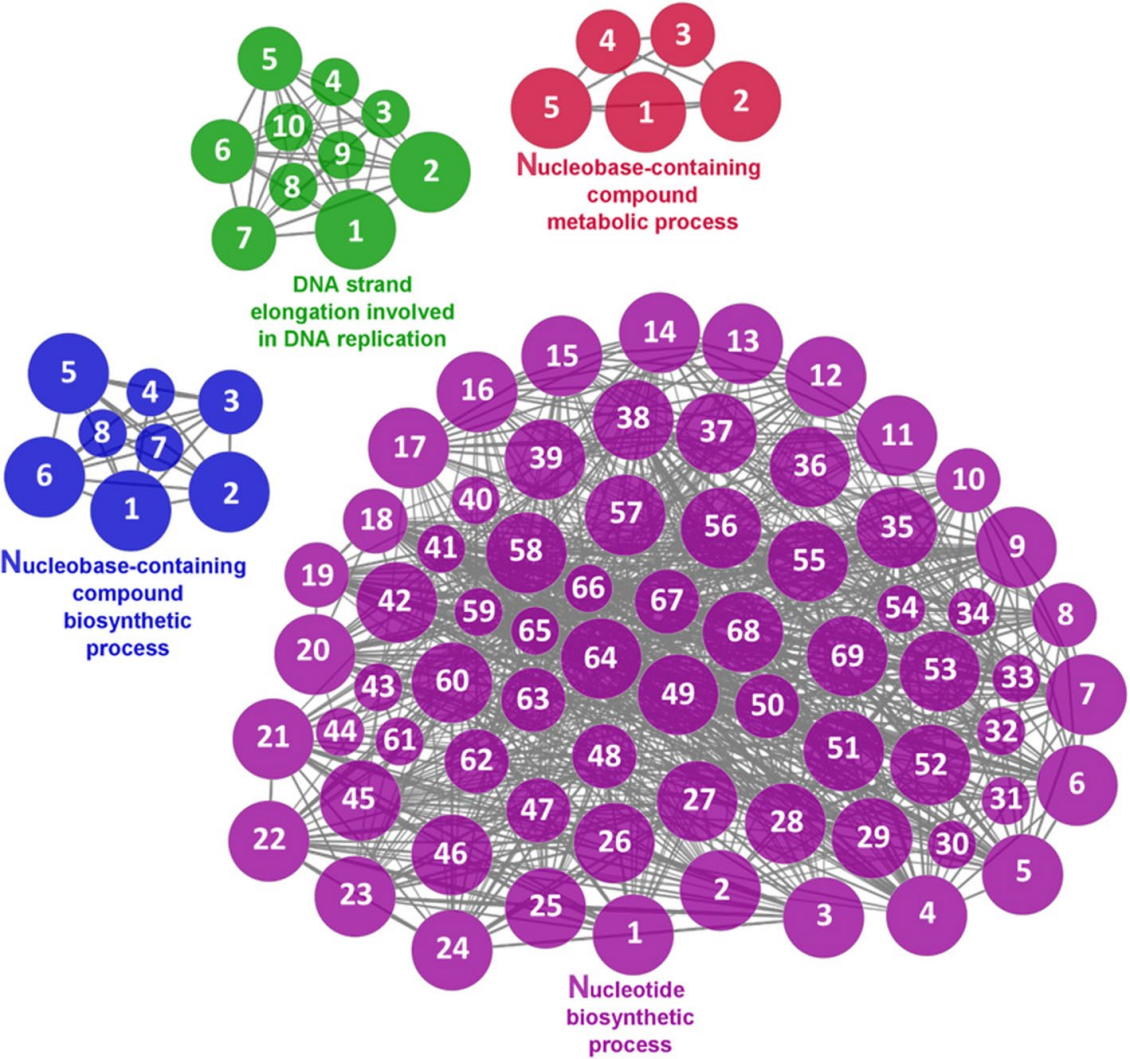
Nucleotide metabolism cluster of the functional enrichment network for biological processes based on the related functions of the differentially expressed genes (DEGs) in the anterior-mid intestine of gilthead seabream (*Sparus aurata*) fed experimental low fishmeal diets containing *Debaryomyces hansenii* (1.1%, 17.2 × 10^5^ CFU; Yeast diet) or devoid of the probiotic (Control diet). The node size determinate the significance (Bonferroni step-down correction; small *P* < 0.05; medium *P* < 0.005; large *P* < 0.0005), the node colour determinate the principal GO process aggrupation, and the node numbers indicate different GO process classified in Additional file 1: Table S4

**Fig. 11:**
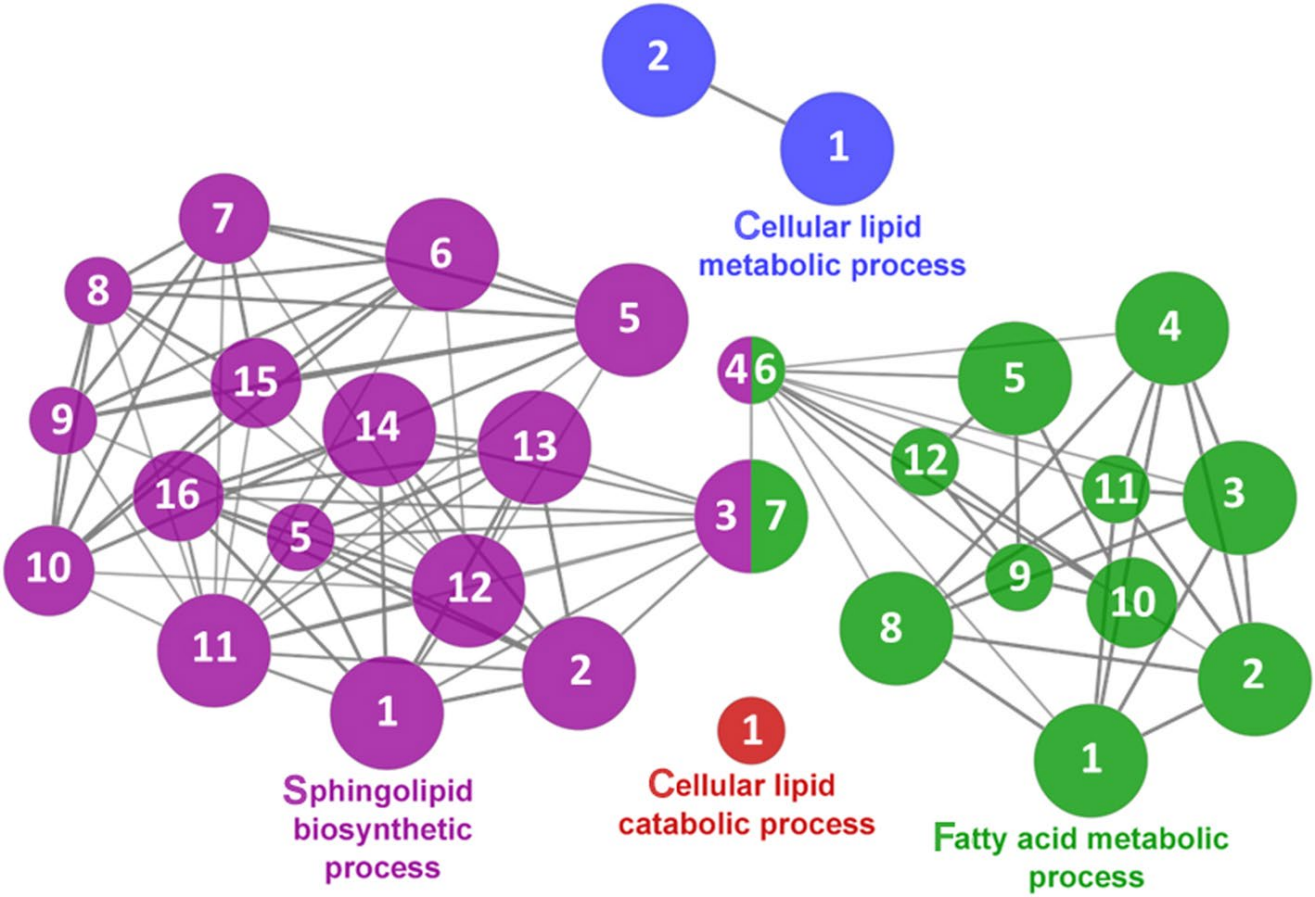
Lipid metabolism cluster of the functional enrichment network for biological processes based on the related functions of the differentially expressed genes (DEGs) in the anterior-mid intestine of gilthead seabream (*Sparus aurata*) fed experimental low fishmeal diets containing *Debaryomyces hansenii* (1.1%, 17.2 × 10^5^ CFU; Yeast diet) or devoid of the probiotic (Control diet). The node size determinate the significance (Bonferroni step-down correction; small *P* < 0.05; medium *P* < 0.005; large *P* < 0.0005), the node colour determinate the principal GO process aggrupation, and the node numbers indicate different GO process classified in Additional file 1: Table S4

**Fig. 12:**
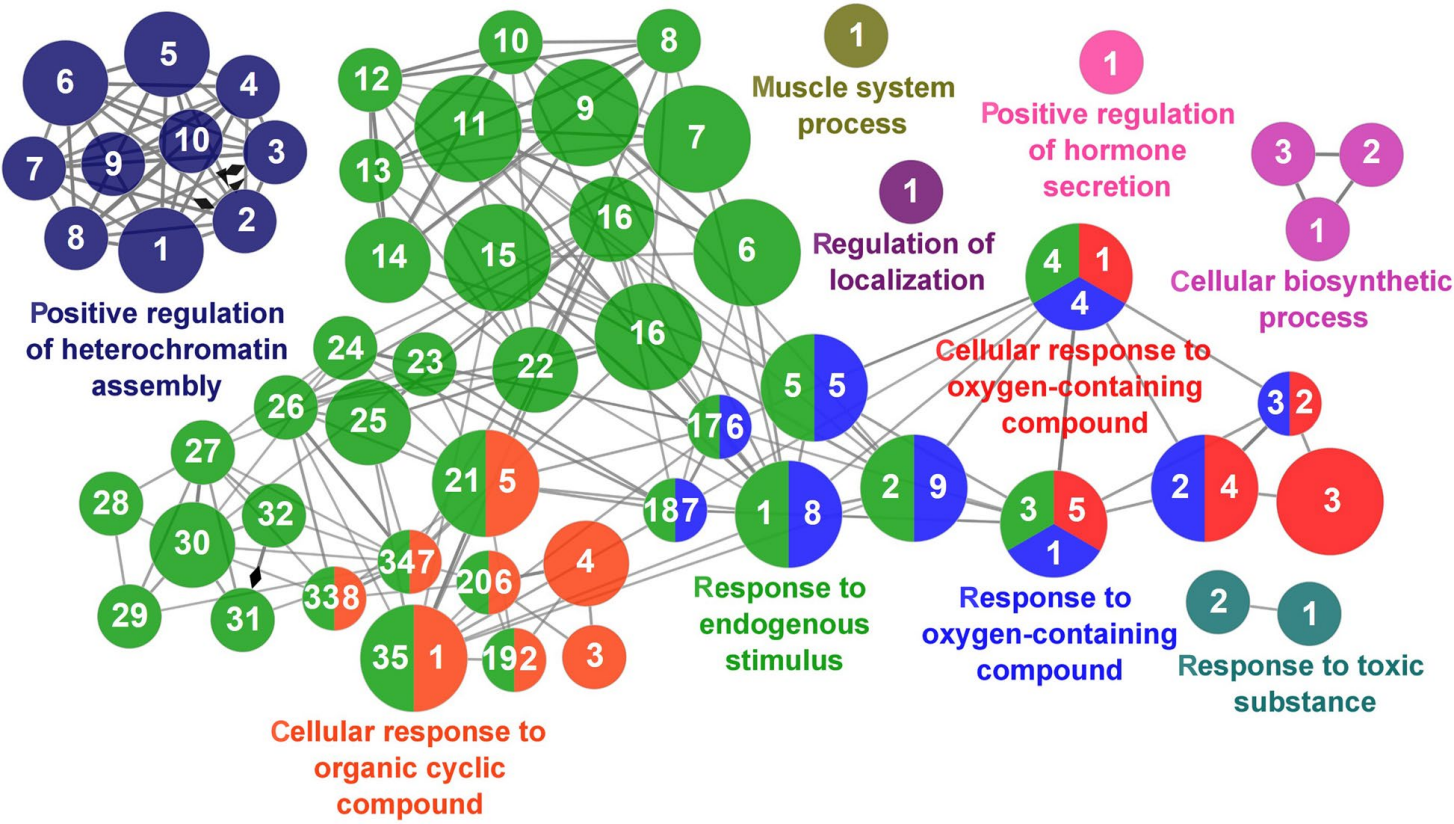
Regulation of Antioxidant Capacity cluster of the functional enrichment network for biological processes based on the related functions of the differentially expressed genes (DEGs) in the anterior-mid intestine of gilthead seabream (*Sparus aurata*) fed experimental low fishmeal diets containing *Debaryomyces hansenii* (1.1%, 17.2 × 10^5^ CFU; Yeast diet) or devoid of the probiotic (Control diet). The node size determinate the significance (Bonferroni step-down correction; small *P* < 0.05; medium *P* < 0.005; large *P* < 0.0005), the node colour determinate the principal GO process aggrupation, and the node numbers indicate different GO process classified in Additional file 1: Table S4

**Fig. 13:**
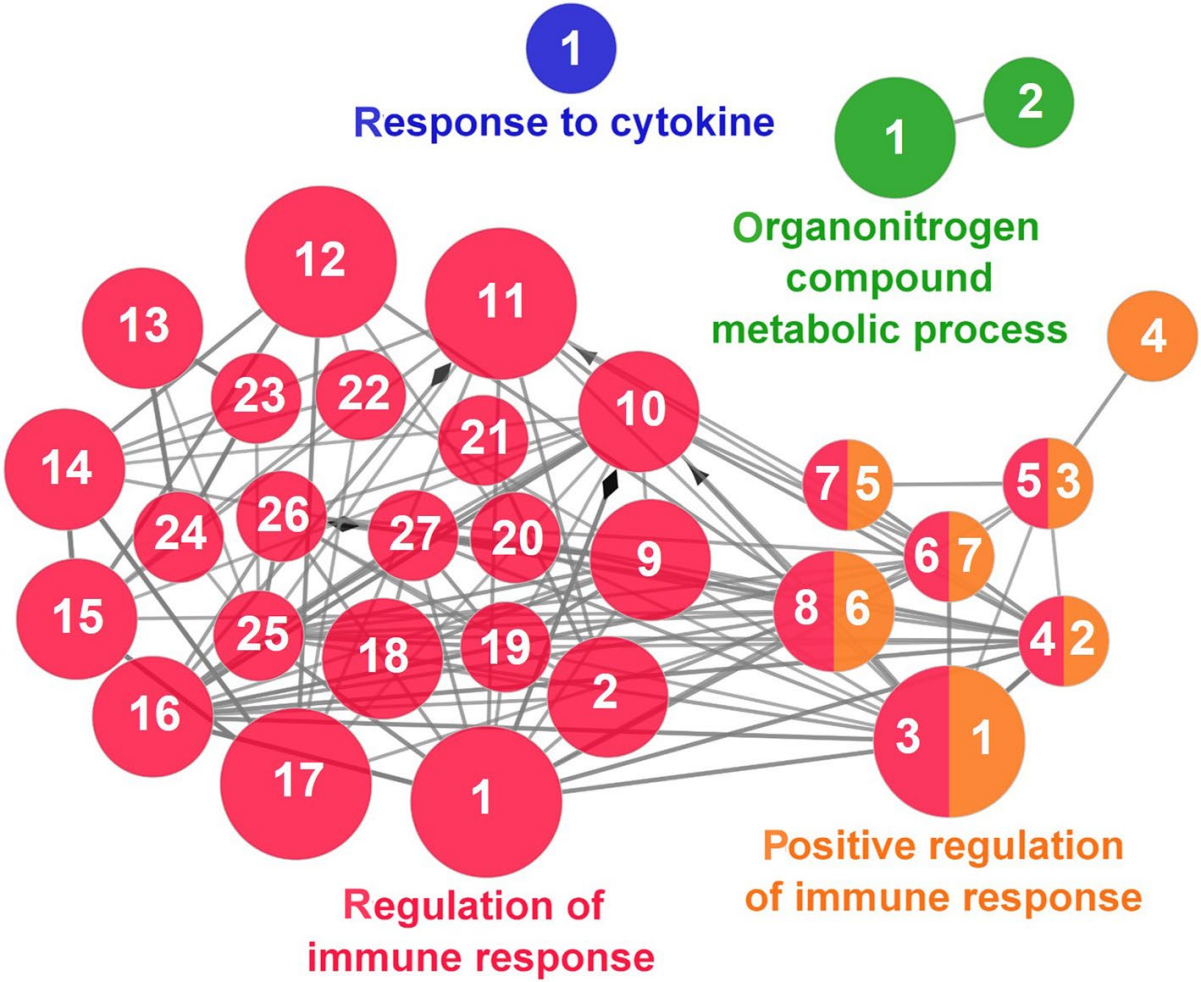
Regulation of Immune Response cluster of the functional enrichment network for biological processes based on the related functions of the differentially expressed genes (DEGs) in the anterior-mid intestine of gilthead seabream (*Sparus aurata*) fed experimental low fishmeal diets containing *Debaryomyces hansenii* (1.1%, 17.2 × 10^5^ CFU; Yeast diet) or devoid of the probiotic (Control diet). The node size determinate the significance (Bonferroni step-down correction; small *P* < 0.05; medium *P* < 0.005; large *P* < 0.0005), the node colour determinate the principal GO process aggrupation, and the node numbers indicate different GO process classified in Additional file 1: Table S4

According to the results reported by the enrichment analysis, representative clusters of genes were classified as shown in Figs. 9, 10, 11, 12 and 13 and Additional file 1: Table S4**.** A significant number of genes were observed to be shared among the metabolic category: Protein Metabolism (31.2% of known genes; Fig. 9), Nucleotide Metabolism (8.3% of known genes; Fig. 10), and Lipid Metabolism (5.7% of known genes; Fig. 11). The other clustered categories contained genes related to the Regulation of Antioxidant Capacity (6.4% of known genes; Fig. 12), and to the Regulation of Immune Response (10.8% of known genes; Fig. 13). This clusters suggest a strong relationship between processes that favours metabolic and immune responses to the dietary inclusion of *D. hansenii*.

Three significative cellular components GO groups (80.2% of the total DEGs in GO:0043227; 73.9% in GO:0043231; 10.2% in GO:0005740) were associated with the single or double lipid bilayer membrane, which indicated that most of DEGs belong to the above-mentioned structures. On the other hand, it is also worth noting that some of these DEGs participate in mitochondrial processes (GO:0005739), proteasome complex (GO:0000502) and DNA replication (replication fork: GO:0005657; replisome: GO:0030894). For further details see Additional file 1: Table S5.

## Discussion

Low fishmeal along with high plant-content meal diets are of common practices for the aquaculture feed industry. However, reducing the dependence on marine raw materials is still associated with variable side effects on fish nutrient metabolism, mucosal health, and disease resistance [42], especially due to the presence of antinutritional factors and the lack of essential amino acids and fatty acids in those alternative sources. Thus, it is necessary to develop complementary nutritional tools to reduce the negative-side effects associated with the substitution of traditional ingredients. In this sense, the use of functional feeds seems to be an appropriate strategy [43]. When considering new additives, yeasts are among those promising candidates showing a wide range of benefits on animal health and growth [21, 44]. In this sense, the present study aimed to determine the effects on growth and intestinal condition of a functional feed with a low fishmeal formula (7%) that was supplemented with *D. hansenii* in *S. aurata*, the main marine fish species farmed in the Mediterranean basin [30].

### Effects of *D. hansenii* on fish performance

Under present experimental conditions, we found a significant body weight increase in fish fed the Yeast diet coupled to a lower feed conversion rate and similar feed intake values compared with those fish fed the Control diet. Considering that feeding costs represent up to 50%–70% of total production costs in intensive fish farms [45], and fishmeal being amongst the most expensive raw materials in aquafeeds [46]; the nutritional strategy tested in our study that consisted of combining a diet containing low fishmeal levels (7%) with the supplementation of a live probiotic seemed very promising when KPIs related to growth and feed utilization were considered. Furthermore, these results are of special relevance since this is the first study in fish juveniles showing the benefits of supplementing low fish meal diets with *D. hansenii* in terms of somatic growth and feed efficiency parameters. In this context, several previous studies had reported that the dietary inclusion of *D. hansenii* (intact or hydrolysed) in aquafeeds with high levels of fishmeal (39%–41%) did not result in significant differences in terms of fish growth performance in Atlantic salmon (*Salmo salar*) and tropical gar (*Atractosteus tropicus*) [27, 47]. Present findings when using low fishmeal diets may be due to the presence in *D. hansenii* of growth factors like biogenic amines, capable of promoting growth and intestinal functionality [21, 29, 48]. Biogenic amines or polyamines are essential metabolites found in almost all living organisms [49]. In particular, *D. hansenii* is especially rich in putrescine, spermidine, spermine and cadaverine [17, 50]. These polyamines are essential for many cell functions and play a key role in vertebrate development and cell growth [17, 51]. In particular, Tovar-Ramírez et al. [25] observed in European sea bass (*Dicentrarchus labrax*) an increase of larval survival and an enhancement of the digestive function, whereas Teles et al. [23] found that longfin yellowtail (*Seriola rivoliana*) larvae had higher growth and survival rates, and an earlier digestive tract maturation when fed with a diet supplemented with *D. hansenii*. These results found in fish larvae were similar to those found in the current study regardless of the developmental stage considered. Furthermore, although it is described that polyamines in high concentrations can generate adverse effects on animal health, their probiotic administration through *D. hansenii* in feed might be of great benefit to fish as reported in several studies [21, 25, 29, 48]. In addition, by participating in the protein metabolism, these polyamines could be of special interest in carnivorous species with high protein demand [51], since their dietary provision by *D. hansenii* may modulate the amino acid metabolism in fish, which could result in the promotion of fish growth by a better use of dietary proteins [52], as shown in our study.

### Effect of *D. hansenii* diet on intestinal condition

The intestine is a complex organ composed of the digestive epithelium with its typical tissue organization in different mucosal layers, its associated lymphoid tissue, and the mucus layer covering the simple intestinal epithelium with its commensal microbiota. Furthermore, enterocytes form a selectively permeable barrier for water, electrolytes, and nutrients, while maintaining an effective defence against pathogens and tolerance towards dietary antigens [53]. Thus, studying the effect of the diet on tissue condition and its commensal microbiota is of importance when evaluating new nutritional strategies. In this context, the intestinal microbiota has been extensively studied in a multitude of fish species since microbial gut communities are essential for proper gastrointestinal development and are implicit in aiding digestive and immunological functions and disease resistance [54, 55]. However, how host-microbiota symbiosis and/or how feed and feed additives can modulate the normal microbiota in the host remains poorly understood, since gut microbial communities are strongly affected by dietary or environmental factors [56, 57]. Despite that, results in terms of phyla obtained in our study were in agreement within those from other authors [40, 58, 59]. In those studies, Proteobacteria has been reported as the most abundant phylum in the intestine of *S. aurata*, followed by Firmicutes, Actinobacteria, and Bacteroidetes. This conservative pattern at phyla level of microbial communities in the intestine was maintained in our study regardless of the diet evaluated.

At a lower taxonomic level, *Pseudomonas* spp. and *Acinetobacter* spp. were the dominant genera in the gut microbiome from both dietary groups. *Pseudomonas* spp. and *Acinetobacter* spp. are a part of the typical fish microbiota [60], whereas some strains have been reported as antagonistic against pathogenic microorganisms [61, 62]. However, under stressful conditions such as malnutrition and overcrowding their abundance and the presence of some opportunistic and pathogenic species may increase [63–65]. Under current experimental condition, the reduction in the relative abundance of these genera in fish fed the Yeast diet suggested that the dietary administration of *D. hansenii* may be a strategy for dealing with infections caused by opportunistic and antimicrobial-resistant bacteria, such as *Pseudomonas* spp. and *Acinetobacter* spp. [66–70]. Furthermore, *D. hansenii* supplementation also resulted in an increase of the relative abundances of ASVs related to the genus *Bacillus* in comparison to fish fed the Control diet. This Gram-positive endospore-forming genus has been widely tested as a probiotic on several aquatic cultured organisms, enhancing growth, modulating immune response, and promoting disease resistance [71, 72]. Similar to other species, *D. hansenii* are normally found as cohabitants in the gut of wild and farmed fish [21, 73], and seems to interact with the host, maintaining intestine microbiota homeostasis, and aiding to its recover in case of dysbiosis [74, 75]. Although in the current study, the low fishmeal diet did not result in any dietarily-induced inflammatory disorder of the intestinal mucosa or dysbiosis of the gut microbiome, these interactions in terms of DEGs related to membrane-linked organelles and symbiotic biological processes were identified by the transcriptomic functional analysis conducted, and further discussed in "Effect of *D. hansenii* diet on gut metabolism, homeostasis, and immunity".

The mucus layer that covers enterocytes is considered as the first line of innate host defence as well as the site where commensal microbiota attaches, preventing the colonisation of pathogenic organisms by occupying empty niches. The dynamic relationship between commensal bacteria and mucins secreted by goblet cells plays an important role in gut health and condition [76]. Commensal microbiota is adapted to the glycan rich environment of mucus, stablishing host-microbiota interactions, influencing on the quantitatively and qualitatively mucin glycosylation [77]. In the current study, the dietary administration of *D. hansenii* did not modify the histochemical properties of mucins produced by goblet cells in terms of neutral glycoproteins, but resulted in a slightly higher richness of carboxylated and weakly ionised sulphated glycoconjugates in fish fed the Yeast diet. These results are of special relevance since the pattern of O-glycosylation in mucin glycoproteins and their polypeptide backbone structure linked to some physical properties of the mucus layer as its viscosity and depth, are key elements in gut health and condition modulation [78]. Thus, the increase in carboxylated and weakly ionized acid sulphated grlycoproteins produced by goblet cells in fish fed the Yeast diet may be interpreted as beneficial due to an increase in mucus viscosity that would protect the host form opportunistic pathogenic bacterial [79, 80]. Furthermore, the inclusion of *D. hansenii* in the diet changed the glycosylation patterns of intestinal mucins, being of special relevance the increase in SBA lectin affinity rather than WGA, changes that may be partially associated to different rates of mucin glycosylation and goblet cell differentiation along the intestinal mucosa [34]. However, these results are of special relevance since lectins with affinity for SBA, which bind to GalNAc residues may contain sialic acid residues in the backbone region of the mucin [81], have been postulated to have an antiparasitic effect in turbot (*Scophthalmus maximus*) [82] and pathogenic organisms [78, 83]. Furthermore, the above-mentioned changes in lectin affinity may be supported by differential expression of *b4galt3* and *b3galt2* genes, which both participates in the elongation and branching biosynthetic steps of glycosylation pathways [84], and the up-expression of a Golgi mannosyl-oligosaccharide 1,2-α-mannosidases gene (*man1a1*), whose activity is a prerequisite for the formation of complex or hybrid glycans of mucins [85].

Overall, the results obtained showed the ability of *D. hansenii* to modulate the gut microbiota without affecting the intestine cell organization in *S. aurata*, mainly reducing several groups of Proteobacteria abundances, especially those opportunistic groups, and changing glycosylation patterns of intestinal mucins. It is suggested that these results, which can be attributed to the potential role of *D. hansenii* as a probiotic, involve some of the yeast cell wall components, such as β-glucan, polyamines and mannan molecules that have, apart from these attributes, the ability to modulate the response of the immune system [21, 86, 87]. However, the functional benefits of these findings need further attention, including the role of *D. hansenii* and the microbiota or their interactions, in terms of glycosylation patterns variation.

### Effect of *D. hansenii* diet on gut metabolism, homeostasis, and immunity

In parallel with the improved performance and the modulation of the intestinal condition, several authors have suggested that the dietary administration of *D. hansenii* result in a concomitant modification of systemic physiological responses, and especially those related to the digestive function and immunity [17, 88]. In this sense, the dietary inclusion of *D. hansenii* modulated the expression of different genes in the anterior-mid intestine of *S. aurata* that were associated with biological processes related to gut metabolism, antioxidant defence, and immunity.

Compared with terrestrial animals, fish have higher protein requirements due to their biological lifestyle characteristics [89]. For this reason, it is not surprising that most of the DEGs with metabolic functions were mainly related to protein biosynthesis, turnover and degradation to cope with their increased growth rate. In our study, we found that the ubiquitin–proteasome system (UPS) was modulated by the regulation of some ubiquitin enzyme and ligases genes (*uchl3*, *trim 25*, *trim39*, *ubr7* and *ube2i*) and proteasome subunits (*psma4*, *psma6*, *psmb10*, *psmc2* and *rad23a*), which act as protein binding signalling and protein degradation, respectively. Both activities are suggested to be important mechanisms of aging, immune activity, cell quality control and protection, tissue regeneration, or DNA repair and gene transduction [90, 91]. On the other hand, we found several DEGs related to protein synthesis and modification, especially genes involved in ribosomal subunit components (*rpl22*, *rpl38*, *mrps2*, *mrps9*, and *mrpl54*), and amide-related biosynthetic processes. The regulation of several genes involved in amide biosynthesis may be attributed to the amine and polyamine content of *D. hansenii* included in the diet [17], which would support the above-mentioned benefits in terms of growth performance as described in several species [21].

Linked to the increased gut metabolic activity, we found other DEGs not directly related to protein metabolism that were associated with nucleotide metabolism and DNA elongation and repair. According to current results, dietary *D. hansenii* enhanced thymidylate nucleotide metabolism as indicated by the up-regulation of *dtymk*, *tyms*, *dut*, and *dck* genes. Thymidylate is necessary to synthetise deoxythymidine triphosphate (dTTP), an essential building block of DNA [92]. In particular, studies in larvae showed that TYMK, the protein codded by *dtymk* gene, and its deoxyribonucleotide triphosphate (dNTP) product, are vital in early embryogenesis and essential for neurodevelopment, and genome integrity in adult fish [93, 94]. These results would be in agreement with the reported beneficial effect of *D. hansenii* larval development and morphogenesis [23, 25].

The third main metabolic cluster identified in our study was related to lipid metabolism, mainly involving DEGs related to sphingolipid or ceramide metabolic processes (*aldh3a2*, *elovl6*, *asah1*, *degs1*, *b3galt2* and *b4galt3*). Sphingolipids and ceramides are constituents of eucaryotic cell membranes with a wide variety of functions and properties. It is reported that sphingolipids have antimicrobial and immunomodulatory properties [95], along with bioactive signalling functions [96]. The DEG *asah1* encodes for an acid ceramidase N-acylsphingosine amidohydrolase 1 (ASAH1), which catalyses the degradation of ceramide into sphingosine and free fatty acid and suggested to maintain ceramide homeostasis in the lysosome [97]. Furthermore, participates in sphingosine recycling pathway along with *degs1* gene, which controls the step from dihydroceramides to ceramides, and suggested to be a promising biomarker of redox state [98].

An increase in the metabolism could lead to increased production of oxidative radicals, that in an unbalanced situation with the antioxidant defence capacity would produce oxidative damage [99]. In parallel to the above-mentioned enhancement of gut metabolic activity, we also found an increased expression of some genes from the electron chain and others also involved in mitochondrial respiration. Their activity produces chemical energy for the normal cell behaviour, but as a result the superoxide anion ($\mathrm O_2^-$), the precursor of most other reactive oxygen species (ROS), are also obtained [100]. To possibly counteract with this situation, we found the up-regulation of *mgst3* and *gsto1* genes in fish fed the Yeast diet, which both together are related with the glutathione cycle, in charge of detoxifying hydrogen peroxide (H_2_O_2_) [101], and *selenot*, which mediates oxidoreductase functions involved in redox homeostasis [102]. Magalhães et al. [103] reported in *S. aurata* different antioxidant levels depending on the organ analysed, being the enzymatic activity of catalase (CAT), superoxide dismutase (SOD) and glutathione reductase (GR) higher in the intestine, and glutathione peroxidase (GPx) in liver. The authors discussed that this pattern is expected due to the elevated cell turnover rate in the intestine that favours oxidative processes in a non-stressful situation, which is also proposed in our study by the supposedly increased gut cell metabolism linked to the increased somatic growth observed. As H_2_O_2_ plays important cellular functions, e.g., as a promoter for cell cycle progression, the activity of the glutathione cycle enzymes and cytochrome P450 (*cyp1a1*), also takes part in metabolic pathways like arachidonic, leukotriene, prostaglandin metabolism, or in estrogen metabolism [51, 104], as well as protecting lipids and proteins to be oxidised [105]. In that way, the up-regulation of malate/oxoglutarate carrier (*slc25a11*) that is related with the TCA cycle and also plays a key role maintaining GSH levels in the mitochondria [106], would reinforce this hypothesis. Thus, the up-regulation of these genes could be more related to metabolism than to a possible oxidative damage produced by the inclusion of yeast in the diet. This idea is also supported by some down-regulated genes related to oxidative stress like: *serpinb1*, which is suggested to protect the cell from proteases released into the cytoplasm during stress [107]; *sin3a*, whose expression is promoted under hypoxia stress [108]; and the anti-inflammatory cytokine IL-10, that is modulated under redox/oxidant perturbations [109]. Regarding antioxidant functions, dietary live yeast administration in *D. labrax* larvae, induced lower activity and expression levels of glutathione peroxidase and superoxide dismutase compared to fish fed control diet, assuming a possible involvement of superoxide anion retention in fish larvae, which could represent importance to the host to increase cell or tissue responsiveness to growth- and/or differentiation-enhancing factors [110].

As reviewed in Angulo et al. [21], *D. hansenii* dietary supplementation has been shown to promote gut protection in several aquatic and terrestrial species at immunological and gene expression levels. An exhaustive analysis of the DEGs promoted by the Yeast diet in our study showed different regulatory responses of the *S. aurata* gut immune system. Among them, we found regulatory viral genes up-regulated like the *lsm14a*, a sensor for both viral DNA and RNA which is important to initiate the IFN-β induction [111]; the *trim25* gene, that encodes the TRIM25 E3 ubiquitin ligase protein, which mediates an essential step to initiate the antiviral responses in the cell through the pattern recognition receptor RIG-I [112]; and the *phb2*, a gene that encodes a prohibitin 2 protein related to the mitochondrial-mediated antiviral innate immunity by activating the RIG-I signalling pathway [113]. Yeast dietary inclusion also promoted the up-regulation of regulatory genes required for T and B cell development and proinflammatory mediators (*nsd2*, *rabl3*, *inhbb*, and *parp1*), a lymphocyte activator (*slamf8*), and genes involved in proteasomal generation of antigenic peptides (*psma4* and *psma6*). All these genes might participate in the first steps of the immune response, promoting the regulation of sentinel immune responses, maintaining the immune cells quiescent to avoid autoimmunity, but prepared for a rapid activation [114]. Contrary to those up-regulated genes, we found down-regulated genes directly related with the activation of immune system processes: *serpinb1*, which is suggested to be activated upon an infection [115]; the *tyrobp* gene, which participates in the activation of immune cells involved in inflammatory reactions and as a component of NK cells with anti-viral functions [116]; and the *cfh* gene, which regulates the alternative pathway of the complement system [117]. We also found two cytokines down-regulated, a pro-inflammatory *il15* gene, which promotes Th1 responses and T-cell maturation [118], and *il10*, an anti-inflammatory gene that is expressed under infection with stimulatory and inhibitory activities on several immune cells [119]. Altogether, it may be postulated that yeast supplementation in a low fish meal feed could promote the regulation of sentinel immune processes by increasing the potentiation of the innate immunity rather than activating a response of the immune markers. A good example of this hypothesis was shown by the down-regulation of *psmb10*, which is an IFN-inducible immunoproteasome, mainly activated during an immune response [120]. In that sense, it has been postulated that *D. hansenii* might modulate host’s immunity through its wall-related β-glucan and polyamine content, increasing functional and decreasing deleterious immune responses in fish [121]. How these components act or in which pathways are involved is still poorly studied. However, as β-glucans are considered pathogen-associated molecular patterns, there seems to be a consensus in its effects potentiating cellular responses and gene expression signalling pathways, activating the communication and activity of the adaptive immune system in several marine species [122–124], which could explain the vast response of the intestine at transcriptomic level.

## Conclusions

The present multi-integrative study of the intestinal response to the probiotic *D. hansenii* when administered to *S. aurata* juveniles in diets with low fishmeal levels provided new insights into the use of this yeast and its metabolic and immunomodulatory components on farmed fish. Under current experimental conditions, we demonstrated that dietary administration of *D. hansenii* (1.1%, 17.2 × 10^5^ CFU) enhanced somatic growth and improved feed efficiency parameters, results that were coupled to an improvement of intestinal condition as histochemical, microbiota and transcriptomic tools indicated. In particular, *D. hansenii* stimulated host-microbiota interactions without altering the intestinal cell organization nor generating dysbiosis, which demonstrated the safety use of the yeast as a probiotic. At the transcriptomic level, *D. hansenii* promoted metabolic pathways, mainly protein-related, sphingolipid, and thymidylate pathways, in addition to enhance antioxidant-related intestinal mechanisms, and to regulate sentinel immune processes, potentiating the defensive capacity meanwhile maintaining the homeostatic status of the intestine. These findings confirm the probiotic benefits of *D. hansenii* in the diet, and its use on cultured marine species beyond their larval stages.

## Supplementary Information

**Additional file 1: Fig. S1: **Principal coordinate analysisof the anterior-mid intestine microbiota similarity. **Table S1:** Summary of the differentially expressed genesobtained from the microarray-based transcriptomic analysis for the gut of juvenile gilthead seabream fed the *D. hansenii* diet. **Table S2: **Functional enrichment analysis of the DEGs. Biological processidentified in the intestine of juvenile gilthead seabreamfed the *D. hansenii* diet. **Table S3: **Top-10 biological processof the DEGs identified in the intestine of juvenile gilthead seabreamfed the *D. hansenii* diet. **Table S4: **Representative cluster of the functional enrichment network for biological processes based on the related functions of the differentially expressed genesin the anterior-mid intestine. **Table S5: **Cellular componentand annotated keywordsof the DEGs identified in the intestine of juvenile gilthead seabreamfed the *D. hansenii* diet.

## Data Availability

Detailed information and transcriptomic raw data are available at the Gene Expression Omnibus (GEO) public repository at the US National Center for Biotechnology Information (NCBI), accession numbers GPL13442, and GSE162504, respectively. Data on intestinal microbiota are publicly available in the NCBI SRA depository (SRS16609731—SRS16609735) within BioProject PRJNA914904, with BioSample accession numbers SAMN32318396 and SAMN32318408.

## Abbreviations

AB: Alcian blue
ASAH1: N-acylsphingosine amidohydrolase 1
ASVs: Amplicon sequence variants
BW: Body weight
CAT: Catalase
CFU: Colony-forming unit
*D.** hansenii*: *Debaryomyces hansenii*
DEGs: Differential expressed genes
dNTP: Deoxyribonucleotide triphosphate
dTTP: Deoxythymidine triphosphate
FC: Fold-change
FCR: Feed conversion ratio
FDR: False discovery rate
FI: Feed intake
GEO: Gene Expression Omnibus
GR: Glutathione reductase
GO: Gene Ontology
GPx: Glutathione peroxidase
GSH: Reduced glutathione
HRP: Horseradish peroxidase
IFN-β: Interferon beta
IL-10: Interleukin 10
KEGG: Kyoto Encyclopedia of Genes and Genomes
KPI: Key performance indicators
NCBI: National Center for Biotechnology Information
NK: Natural killer
PAS: Periodic Acid Schiff
PCA: Principal component analysis
PCoA: Principal coordinate analysis
PPI: Protein–protein interaction
RIG-I: Retinoic acid-inducible gene I
*S.** aurata*: *Sparus aurata*
SBA: Soybean agglutinin
SD: Standard deviation
SGR: Specific growth rate
SOD: Superoxide dismutase
TCA: Tricarboxylic acid cycle
TRIM25: Tripartite motif containing 25
UPS: Ubiquitin–proteasome system
WGA: Wheat Germ Agglutinin (*Triticum vulgaris* lectin)
